# Afri-Can Forum 2: Johannesburg, South Africa. 16-18 February 2015

**DOI:** 10.1186/s12879-016-1466-6

**Published:** 2016-07-12

**Authors:** Hillary Mukudu, Neil Martinson, Benn Sartorius, Jenny Coetzee, Janan Dietrich, Kgaugelo Mokgatswana, Rachel Jewkes, Glenda E. Gray, Marylène Dugas, Luc Béhanzin, Fernand A. Guédou, Marie-Pierre Gagnon, Michel Alary, Rwamahe Rutakumwa, Martin Mbonye, Thadeus Kiwanuka, Sarah Nakamanya, Richard Muhumuza, Winfred Nalukenge, Janet Seeley, Millicent Atujuna, Melissa Wallace, Ben Brown, Linda Gail Bekker, Peter A. Newman, Rushil Harryparsad, Abraham J. Olivier, Heather B. Jaspan, Douglas Wilson, Janan Dietrich, Neil Martinson, Hillary Mukudu, Nonhlanhla Mkhize, Lynn Morris, Gianguido Cianci, Minh Dinh, Thomas Hope, Jo-Ann S. Passmore, Clive M. Gray, Bethany M. Henrick, Xiao-Dan Yao, Kenneth L. Rosenthal, the INFANT Study Team, Bethany M. Henrick, Xiao-Dan Yao, Anna G. Drannik, Alash’le Abimiku, Kenneth L. Rosenthal, the INFANT Study Team, Nadia Chanzu, Walter Mwanda, Julius Oyugi, Omu Anzala, Moustapha Mbow, Sabelle Jallow, Moussa Thiam, Alberta Davis, Assane Diouf, Cheikh T. Ndour, Moussa Seydi, Tandakha N. Dieye, Souleymane Mboup, Martin Goodier, Eleanor Rilley, Assan Jaye, Xiao-Dan Yao, RW. Omange, Bethany M. Henrick, Richard T. Lester, Joshua Kimani, T. Blake Ball, Francis A. Plummer, Kenneth L. Rosenthal, Luc Béhanzin, Fernand A. Guédou, Nassirou Geraldo, Ella Goma Mastétsé, Jerôme Charles Sossa, Marcel Djimon Zannou, Michel Alary, Sophia Osawe, Evaezi Okpokoro, Felicia Okolo, Stephen Umaru, Rebecca Abimiku, Sam Audu, Pam Datong, Alash’le Abimiku, Jacquelyn Nyange, Joyce Olenja, Gaudensia Mutua, Walter Jaoko, Gloria Omosa-Manyonyi, Bashir Farah, Maureen Khaniri, Omu Anzala, Anne Cockcroft, Kendra Tonkin, Indu Girish, Puna Mhati, Ashley Cunningham, Neil Andersson, Bashir Farah, Jackton Indangasi, Walter Jaoko, Gaudensia Mutua, Maureen Khaniri, Jacquelyn Nyange, Omu Anzala, Thabo Diphoko, Simani Gaseitsiwe, Victoria Maiswe, Thato Iketleng, Dorcas Maruapula, Keabetswe Bedi, Sikhulile Moyo, Rosemary Musonda, Mark Wainberg, Joseph Makhema, Vladimir Novitsky, Richard Marlink, Max Essex, Stephen Okoboi, Livingstone Ssali, Sam Kalibala, Josephine Birungi, Aggrey Egessa, Jonathan Wangisi, Lyavala Joanne Okullu, Celestin Bakanda, Francis Obare, I. Marion Sumari-de Boer, Hadija H. Semvua, Jossy van den Boogaard, Krisanta W. Kiwango, Kennedy M. Ngowi, Pythia T. Nieuwkerk, Rob E. Aarnoutse, Ireen Kiwelu, Eva Muro, Gibson S. Kibiki, Ruth Datiri, Grace Choji, Sophia Osawe, Evaezi Okpokoro, Felicia Okolo, Stephen Umaru, Rebecca Abimiku, Samuel Audu, Pam Datong, Alash’le Abimiku, A. Fomsgaard, I. Karlsson, K. J. Jensen, S. S. Jensen, C. Leo-Hansen, S. Jespersen, D. Da Silva Té, C. M. Rodrigues, Z. J. da Silva, C. M. Janitzek, J. Gerstoft, G. Kronborg, the WAPHIR Group, Evaezi Okpokoro, Sophia Osawe, Ruth Daitiri, Grace Choji, Stephen Umaru, Felicia Okolo, Pam Datong, Alash’le Abimiku, Nyariki Emily, Olenja Joyce, Lorway R. Robert, Anzala Anzala, Katie Viljoen, Jerome Wendoh, Elvis Kidzeru, Ulas Karaoz, Eoin Brodie, Gerrit Botha, Nicola Mulder, Clive Gray, William Cameron, Alain Stintzi, Heather Jaspan, for the INFANT study team, Paul N. Levett, David Alexander, Naveed Gulzar, Prabvir S. Grewal, Art F. Y. Poon, Zabrina Brumme, P. Richard Harrigan, James I. Brooks, Paul A. Sandstrom, Stryker Calvez, Stephen E. Sanche, Jamie K. Scott, Leslie Swartz, Ashraf Kagee, Anthea Lesch, Zuhayr Kafaar, Anneliese De Wet, Evaezi Okpokoro, Sophia Osawe, Ruth Daitiri, Grace Choji, Stephen Umaru, Felicia Okolo, Pam Datong, Alash’le Abimiku, Janan Dietrich, Tricia Smith, Laura Cotton, Stefanie Hornschuh, Martin van der Watt, Cari L. Miller, Glenda Gray, Jenni Smit, Manjeetha Jaggernath, Thumbi Ndung’u, Mark Brockman, Angela Kaida, on behalf of the AYAZAZI study teams, Maureen Akolo, Joshua Kimani, Larry Gelmon, Michael Chitwa, Justus Osero, Anne Cockcroft, Nobantu Marokoane, Leagajang Kgakole, Boikhutso Maswabi, Neo Mpofu, Umaira Ansari, Neil Andersson, Elizabeth Nakinobe, George Mukalazi Miiro, Flavia Zalwango, Jessica Nakiyingi-Miiro, Potiano Kaleebu, John Ross Semwanga, Emily Nyanzi, Saidat Namuli Musoke, Elizabeth Nakinobe, George Miiro, Edward Katongole Mbidde, Tom Lutalo, Pontiano Kaleebu, Ray Handema, Graham P. Chianzu, Moussa Thiam, Diabou Diagne-Gueye, Mame K. Ndiaye, Moustapha Mbow, Birahim P. Ndiaye, Ibrahima Traore, Mamadou C. Dia, Gilleh Thomas, Coumba Tour-Kane, Souleymane Mboup, Assan Jaye, Emily Nyanzi, Edward Katongole Mbidde, Pontiano Kaleebu, Juliet Mpendo, Joshua Kimani, Josephine Birungi, Winnie Muyindike, Andrew Kambugu, Hachizovu Sebastian, Handema Ray, Chaponda Mike, Kabuya Jean Bertin, Mulenga Modest, Moussa Thiam, Omar Janha, Alberta Davis, Alfred Amambua-Ngwa, Davis C. Nwakanma, Souleymane Mboup, Assan Jaye, Sanne Jespersen, Bo Langhoff Hønge, Joakim Esbjörnsson, Candida Medina, David Da Silva TÉ, Faustino Gomes Correira, Alex Lund Laursen, Lars Østergaard, Andreas Andersen, Peter Aaby, Christian Erikstrup, Christian Wejse, for the Bissau HIV Cohort study group, Siry Dieye, Moussa Sarr, Haby Sy, Helene D. Mbodj, Marianne Ndiaye, Amy Ndiaye, Seydi Moussa, Assan Jaye, Souleymane Mboup, Balthazar M. Nyombi, Elichilia R. Shao, Innocent B. Chilumba, Sikhulile Moyo, Simani Gaseitsiwe, Rosemary Musonda, Pam Datong, Bucky Inyang, Sophia Osawe, Abel Izang, Chundung Cole, Felicia Okolo, Bill Cameron, Kenneth Rosenthal, Clive Gray, Heather Jaspan, Alash’le Abimiku, the INFANT study team, Boitumelo Seraise, Kerstin Andrea-Marobela, Sikhulile Moyo, Rosemary Musonda, Joseph Makhema, Max Essex, Simani Gaseitsiwe

**Affiliations:** Khula Ndoda Mass Male Circumcision Project, Perinatal HIV Research Unit, Chris Hani-Baragwanath Academic Hospital, Johannesburg, South Africa; Perinatal HIV Research Unit, Chris Hani Baragwanath Hospital, Soweto, South Africa; Medical Research Council of South Africa, Cape Town, South Africa; Université Laval, Québec, Canada; Medical Research Council/UVRI Uganda Research Unit on AIDS, Entebbe, Uganda; Desmond Tutu Foundation, University of Cape Town, Cape Town, South Africa; Institute of Infectious Diseases and Molecular Medicine, University of Cape Town, Cape Town, South Africa; Department of Medicine, Edendale Hospital, KwaZulu Natal, South Africa; Perinatal HIV Research Unit, Soweto, South Africa; National Institute for Communicable Diseases, Sandringham, South Africa; Division of Infectious Diseases, Department of Medicine, Northwestern University Feinberg School of Medicine, Chicago, Illinois USA; National Health Laboratory Services, Cape Town, South Africa; McMaster Immunology Research Centre, Michael G. DeGroote Institute for Infectious Disease Research, Department of Pathology & Molecular Medicine, McMaster University, Hamilton, Ontario Canada; McMaster Immunology Research Centre, Michael G. DeGroote Institute for Infectious Disease Research, Department of Pathology & Molecular Medicine, McMaster University, Hamilton, Ontario, Canada; Institute of Human Virology, University of Maryland School of Medicine, Baltimore, Maryland USA; KAVI Institute of Clinical Research, University of Nairobi, Nairobi, Kenya; Laboratory of Bacteriology and Virology of Aristide Le Dantec University Hospital, Dakar, Senegal; Medical Research Council (MRC) Unit, Serrekunda, The Gambia; Clinic of Infectious Disease of Fann University Hospital, Dakar, Senegal; Department of Immunology and Infection, London School of Hygiene & Tropical Medicine, London, UK; McMaster Immunology Research Centre, Michael G. DeGroote Institute for Infectious Disease Research, Department of Pathology & Molecular Medicine, McMaster University, Hamilton, Ontario Canada; Univeristy of Manitoba and Public Health Agency of Canada, Winnipeg, Manitoba Canada; BCCDC, University of British Columbia, Vancouver, British Columbia Canada; Department of Medical Microbiology, University of Nairobi, Nairobi, Kenya; Dispensaire IST, Centre de santé communal de Cotonou 1, Cotonou, Bénin; Centre de recherche du CHU de Québec, Québec, Canada; Programme national de lutte contre le Sida et les IST (PNLS-IST), Cotonou, Bénin; Faculté des sciences de la santé, Université d’Abomey-Calavi, Cotonou, Bénin; Centre national hospitalier universitaire HMK de Cotonou, Cotonou, Bénin; Département de médecine sociale et préventive, Université Laval, Québec, Canada; Institut national de santé publique du Québec, Québec, Canada; Plateau State Human Virology Research Centre (PLASVIREC), Jos, Nigeria; Institute of Human Virology (IHVN), Abuja, Nigeria; Institute of Human Virology, School of Medicine, University of Maryland (UMD), Baltimore, USA; KAVI –Institute of Clinical research, Nairobi, Kenya; CIET Trust, PO Box 1240, Gaborone, Botswana; University of Nairobi, Nairobi, Kenya; Botswana Harvard AIDS Institute Partnership, 1836 Northring Road, Princess Marina Hospital, Gaborone, Botswana; The AIDS Support Organization (TASO), Kampala, Uganda; HIV core/population Council, Washinghton, DC USA; HIV core/population Council, Nairobi, Kenya; Kilimanjaro Clinical Research Institute, Moshi, Tanzania; University of Amsterdam, Amsterdam, The Netherlands; Radboud University, Nijmegen, The Netherlands; Plateau State Human Virology Research Centre (PLASVIREC), Jos, Nigeria; Institute of Human Virology (IHVN), Abuja, Nigeria; Institute of Human Virology, School of Medicine, University of Maryland (UMD), Baltimore, USA; Department of Virology, Statens Serum Institut, Copenhagen, Denmark; The Bandim Health Project, Bissau, Guinea-Bissau; Centro de Tratamiento Ambulatório, Hospital Nacional Simão Mendes, Bissau, Guinea-Bissau; National Laboratory, Bissau, Guinea-Bissau; Department of Infectious Diseases, Rigshospitalet, Copenhagen, Denmark; Department of Infectious Disease Hvidovre Hospital, Hvidovre, Denmark; ᅟ, Senegal; Institute of Human Virology, Abuja, Nigeria; Plateau State Human Virology Research Centre, Jos, Nigeria; Institute of Human Virology, School of Medicine, University of Maryland, Baltimore, USA; University of Nairobi, Nairobi, Kenya; Division of Immunology, Institute of Infectious Disease and Molecular Medicine, University of Cape Town, Cape Town, South Africa; Department of Molecular Biology & Biochemistry, Faculty of Science and Faculty of Health Sciences Simon Fraser University, University Drive Burnaby, Unceded Coast Salish Territories, Canada; Stellenbosch University, Cape Town, South Africa; Institute of Human Virology, Abuja, Nigeria; Plateau State Human Virology Research Centre, Jos, Nigeria; Institute of Human Virology, School of Medicine, University of Maryland, Baltimore, USA; Bio-Behavioural Research, Perinatal HIV Research Unit, Soweto, South Africa; Faculty of Health Sciences, Simon Fraser University, British Columbia, Canada; MatCH Research, University of the Witwatersrand, Durban, South Africa; HIV Pathogenesis Programme, University of KwaZulu-Natal, Durban, South Africa; Partners in Health and Development in Africa, Nairobi, Kenya; University of Nairobi/ University of Manitoba-Nairobi, Nairobi, Kenya; Kenyatta University-Nairobi, Nairobi, Kenya; CIET Trust, Gaberone, Botswana; Uganda Virus Research Institute Entebbe, Entebbe, Uganda; MRC/UVRI Uganda Research Unit on AIDS Entebbe, Entebbe, Uganda; Uganda Virus Research Institute, Entebbe, Uganda; MRC/UVRI Uganda Research Unit on AIDS, Entebbe, Uganda; Rakai Health Sciences program, Entebbe, Uganda; Tropical Diseases Research Centre, Ndola, Zambia; Laboratory of Bacteriology-Virology, Le Dantec Hospital of Dakar, Senegal and Cheikh Anta DIOP University of Dakar, Dakar, Senegal; Social Hygiene Institute of Dakar, Dakar, Senegal; Medical Research Council, Fajara Unit, Banjul, The Gambia; Uganda Virus Research Institute, Entebbe, Uganda; MRC/UVRI Uganda Research Unit on AIDS, Entebbe, Uganda; UVRI-International Aids Vaccine Initiative, Entebbe, Uganda; Kenya AIDS Control Project, University of Nairobi, Nairobi, Kenya; The Aids Support Organization (TASO), Walay city, Uganda; Mbarara University of Science and Technology, Mbarara, Uganda; Infectious Disease Institute, Mulago, Kampala Uganda; Tropical Disease Research Centre, Ndola, Zambia; Bacteriology and Virology Laboratory, Le Dantec Hospital of Dakar, Senegal and Cheikh Anta DIOP University of Dakar, Dakar, Senegal; Medical Research Council, Fajara Unit, Banjul, The Gambia; Bandim Health Project, Indepth Network, Bissau, Guinea-Bissau; Department of Infectious Diseases, Aarhus University Hospital, Aarhus, Denmark; Department of Experimental Medical Science, Section of Molecular Virology, Lund University, Lund, Sweden; Weatherall Institute of Molecular Medicine, University of Oxford, Oxford, United Kingdom; National HIV Programme, Ministry of Health, Bissau, Guinea-Bissau; Research Center for Vitamins and Vaccines (CVIVA), Bandim Health Project, Statens Serum Institut, Copenhagen, Denmark; Department of Clinical Immunology, Aarhus University Hospital, Aarhus, Denmark; GloHAU, Center for Global Health, School of Public Health, Aarhus University, Aarhus, Denmark; Centre de Recherche Clinique et de Formation (CRCF) Infectious Diseases Dept, Fann Hospital, Dakar, Sénégal; Laboratory of Bacteriology and Virology, Le Dantec Hospital, Dakar, Senegal; Westat, Rockville, MD USA; Albert Royer Children’s Hospital, Fann Hospital, Dakar, Senegal; Medical Research Council (MRC), Banjul, The Gambia; Kilimanjaro Clinical Research Institute, Moshi, Tanzania; Plateau State Specialist Hospital, Walay city, Nigeria; Plateau State Human Virology Research Center, Institute of Human Virology, Walay city, Nigeria; Divisions of Infectious Diseases and Respirology, University of Ottawa at The Ottawa Hospital, Ottawa, Canada; Department of Pathology & Molecular Medicine, McMaster University, Hamilton, Ontario Canada; Institute of Infectious Diseases and Molecular Medicine, Faculty of Health Sciences, University of Cape Town, Cape Town, South Africa; Institute of Human Virology, University of Maryland School of Medicine, Baltimore, Maryland USA; Department of Biological Sciences, University of Botswana, Gaborone, Botswana; Botswana Harvard AIDS Institute Partnership, Gaborone, Botswana; Harvard School of Public Health, Department of Immunology and Infectious Diseases, Boston, USA

## Abstract

A1 Introduction to the 2nd synchronicity forum of GHRI/CHVI-funded Canadian and African HIV prevention and vaccine teams

O1 Voluntary medical male circumcision for prevention of heterosexual transmission of HIV in adult males in Soweto: What do indicators and incidence rate show?

Hillary Mukudu, Neil Martinson, Benn Sartorius

O2 Developing a peer-led community mobilization program for sex workers in Soweto: HIV risk and demographics

Jenny Coetzee, Janan Dietrich, Kgaugelo Mokgatswana, Rachel Jewkes, Glenda E. Gray

O3 Salient beliefs about adherence: A qualitative survey conducted as part of the demonstration study on "treatment as prevention" (TasP) and "pre-exposure prophylaxis" (PrEP) among female sex workers (FSWS) in Cotonou, Benin

Marylène Dugas, Luc Béhanzin, Fernand A. Guédou, Marie-Pierre Gagnon, Michel Alary

O4 Relative perception of risk as a driver of unsafe sexual practices among key populations: Cases of fisherfolk and women and their partners involved in multiple sexual partnerships in Uganda

Rwamahe Rutakumwa, Martin Mbonye, Thadeus Kiwanuka, Sarah Nakamanya, Richard Muhumuza, Winfred Nalukenge, Janet Seeley

O5 Exploring the acceptability of new biomedical HIV prevention technologies among MSM, adolescents and heterosexual adults in South Africa

Millicent Atujuna, Melissa Wallace, Ben Brown, Linda Gail Bekker, Peter A. Newman

O6 HIV-susceptible target cells in foreskins after voluntary medical male circumcision in South Africa

Rushil Harryparsad, Abraham J. Olivier, Heather B. Jaspan, Douglas Wilson, Janan Dietrich, Neil Martinson, Hillary Mukudu, Nonhlanhla Mkhize, Lynn Morris, Gianguido Cianci, Minh Dinh, Thomas Hope, Jo-Ann S. Passmore, Clive M. Gray

O7 HIV-1 proteins activate innate immune responses via TLR2 heterodimers

Bethany M. Henrick, Xiao-Dan Yao, Kenneth L. Rosenthal, the INFANT Study Team

O8 Characterization of an innate factor in human milk and mechanisms of action against HIV-1

Bethany M. Henrick, Xiao-Dan Yao, Anna G. Drannik, Alash’le Abimiku, Kenneth L. Rosenthal, the INFANT Study Team

O9 Secretor status and susceptibility to HIV infections among female sex workers in Nairobi, Kenya

Nadia Chanzu, Walter Mwanda, Julius Oyugi, Omu Anzala

O10 Natural Killer cell recall responsiveness to Gag-HIV-1 peptides of HIV-1 exposed but uninfected subjects are associated with peripheral CXCR6+ NK cell subsets

Moustapha Mbow, Sabelle Jallow, Moussa Thiam, Alberta Davis, Assane Diouf, Cheikh T. Ndour, Moussa Seydi, Tandakha N. Dieye, Souleymane Mboup, Martin Goodier, Eleanor Rilley, Assan Jaye

O11 Profiles of resistance: Local innate mucosal immunity to HIV-1 in commercial sex workers

Xiao-Dan Yao, RW. Omange, Bethany M. Henrick, Richard T. Lester, Joshua Kimani, T. Blake Ball, Francis A. Plummer, Kenneth L. Rosenthal

O12 Early antiretroviral therapy and pre-exposure prophylaxis for HIV prevention among female sex workers in Cotonou, Benin: A demonstration project

Luc Béhanzin, Fernand A. Guédou, Nassirou Geraldo, Ella Goma Mastétsé, Jerôme Charles Sossa, Marcel Djimon Zannou, Michel Alary

O13 Building capacity for HIV prevention trials: Preliminary data from a Nigerian cohort of HIV exposed sero-negatives (HESN)

Sophia Osawe, Evaezi Okpokoro, Felicia Okolo, Stephen Umaru, Rebecca Abimiku, Sam Audu, Pam Datong, Alash’le Abimiku

O14 Equipping healthcare professionals with skills required for the conduct of clinical trials in an effort to build capacity. Lessons learned

Jacquelyn Nyange, Joyce Olenja, Gaudensia Mutua, Walter Jaoko, Gloria Omosa-Manyonyi, Bashir Farah, Maureen Khaniri, Omu Anzala

O15 Educational technology to support active learning for HIV researchers and planners

Anne Cockcroft, Kendra Tonkin, Indu Girish, Puna Mhati, Ashley Cunningham, Neil Andersson

O16 From Lake Kivu (Rwanda) and Lake Malawi (Tanzania) to the shores of Lake Victoria (Uganda): Strengthening laboratory capacity through Good Clinical Laboratory Practice training

Bashir Farah, Jackton Indangasi, Walter Jaoko, Gaudensia Mutua, Maureen Khaniri, Jacquelyn Nyange, Omu Anzala

O17 Rilpivirine and etravirine resistance mutations in HIV-1 subtype C infected patients on a non-nucleoside reverse transcriptase inhibitor-based combination antiretroviral therapy in Botswana

Thabo Diphoko, Simani Gaseitsiwe, Victoria Maiswe, Thato Iketleng, Dorcas Maruapula, Keabetswe Bedi, Sikhulile Moyo, Rosemary Musonda, Mark Wainberg, Joseph Makhema, Vladimir Novitsky, Richard Marlink, Max Essex

O18 From home-based HIV testing to initiation of treatment: The AIDS Support Organization (TASO) Experience with Home-based HIV Counselling and Testing (HBHCT) among Adolescents in Uganda, 2005-2011

Stephen Okoboi, Livingstone Ssali, Sam Kalibala, Josephine Birungi, Aggrey Egessa, Jonathan Wangisi, Lyavala Joanne Okullu, Celestin Bakanda, Francis Obare^41^

O19 Feasibility study on using real time medication monitoring among HIV infected and Tuberculosis patients in Kilimanjaro, Tanzania

I. Marion Sumari-de Boer, Hadija H. Semvua, Jossy van den Boogaard, Krisanta W. Kiwango, Kennedy M. Ngowi, Pythia T. Nieuwkerk, Rob E. Aarnoutse, Ireen Kiwelu, Eva Muro, Gibson S. Kibiki

O20 Deaths still among sero-discordant cohort in Nigeria despite Access to treatment

Ruth Datiri, Grace Choji, Sophia Osawe, Evaezi Okpokoro, Felicia Okolo, Stephen Umaru, Rebecca Abimiku, Samuel Audu, Pam Datong, Alash’le Abimiku

O21 Therapeutic HIV-1 vaccine trials in Denmark and Guinea-Bissau

Fomsgaard A, Karlsson I, Jensen KJ, Jensen SS, Leo-Hansen C, Jespersen S, Da Silva Té D, Rodrigues CM, da Silva ZJ, Janitzek CM, Gerstoft J, Kronborg G, the WAPHIR Group

O22 Willingness to participate in a HIV vaccine Trial among HIV exposed sero-negative (HESN) persons in Jos, Nigeria

Evaezi Okpokoro, Sophia Osawe, Ruth Daitiri, Grace Choji, Stephen Umaru, Felicia Okolo, Pam Datong, Alash'le Abimiku

O23 Clinical research volunteers’ perceptions and experiences of screening for enrolment at KAVI-Institute of Clinical Research, Kenya

Nyariki Emily, Olenja Joyce, Lorway R. Robert, Anzala Anzala

O24 Gut microbiome, HIV-exposure, and vaccine responses in South African infants

Katie Viljoen, Jerome Wendoh, Elvis Kidzeru, Ulas Karaoz, Eoin Brodie, Gerrit Botha, Nicola Mulder, Clive Gray, William Cameron, Alain Stintzi, Heather Jaspan, for the INFANT study team

O25 Analysis of HIV pol diversity in the concentrated HIV epidemic in Saskatchewan

Paul N. Levett, David Alexander, Naveed Gulzar, Prabvir S. Grewal, Art F. Y. Poon, Zabrina Brumme, P. Richard Harrigan, James I. Brooks, Paul A. Sandstrom, Stryker Calvez, Stephen E. Sanche, Jamie K. Scott

P1 Evaluating a HIV vaccine research community engagement programme at two HIV prevention research centres in the Western Cape

Leslie Swartz, Ashraf Kagee, Anthea Lesch, Zuhayr Kafaar, Anneliese De Wet

P2 Validating HIV acquisition risk score using a cohort HIV exposed sero-negative persons in a discordant relationship in Jos, Nigeria, West Africa

Evaezi Okpokoro, Sophia Osawe, Ruth Daitiri, Grace Choji, Stephen Umaru, Felicia Okolo, Pam Datong, Alash'le Abimiku

P3 Bridging the gap between adults and adolescents and youth adults (AYA) – Employing a youth-centred approach to investigate HIV risk among AYA in Soweto and Durban, South Africa

Janan Dietrich, Tricia Smith, Laura Cotton, Stefanie Hornschuh, Martin van der Watt, Cari L. Miller, Glenda Gray, Jenni Smit, Manjeetha Jaggernath, Thumbi Ndung’u, Mark Brockman, Angela Kaida, on behalf of the AYAZAZI study teams

P4 Neighbours to sex workers: A key population that has been ignored

Maureen Akolo, Joshua Kimani, Prof Larry Gelmon, Michael Chitwa, Justus Osero

P5 Young women’s access to structural support programmes in a district of Botswana

Anne Cockcroft, Nobantu Marokoane, Leagajang Kgakole, Boikhutso Maswabi, Neo Mpofu, Umaira Ansari, Neil Andersson

P6 Voices for action from peri-urban Ugandan students, teachers and parents on HIV/STI prevention: Qualitative research results

Nakinobe Elizabeth, Miiro George Mukalazi, Zalwango Flavia, Nakiyingi-Miiro Jessica, Kaleebu Potiano

P7 Engaging Social Media as an education tool on the fly: The use of Facebook for HIV and Ebola prevention and awareness amongst adolescents in Uganda

John Ross Semwanga, Emily Nyanzi, Saidat Namuli Musoke, Elizabeth Nakinobe, George Miiro, Edward Katongole Mbidde, Tom Lutalo, Pontiano Kaleebu

P8 Circulating HIV-1 subtypes among sexual minority populations in Zambia

Ray Handema, Graham P. Chianzu

P9 The Development of HIV Bio-bank resource management to support clinical trial and Intervention research: WAPHIR experience

Moussa Thiam, Diabou Diagne-Gueye, Mame K. Ndiaye, Moustapha Mbow, Birahim P. Ndiaye, Ibrahima Traore, Mamadou C. Dia, Gilleh Thomas, Coumba Tour-Kane, Souleymane Mboup, Assan Jaye

P10 Capacity building for clinical trials as a novel approach for scaling up HIV prevention research initiatives in East Africa: achievements and challenges

Emily Nyanzi, Edward Katongole Mbidde, Pontiano Kaleebu, Juliet Mpendo, Joshua Kimani, Josephine Birungi, Winnie Muyindike, Andrew Kambugu

P11 Community and media perspective of research; an advocacy workshop on HIV prevention research

Hachizovu Sebastian, Handema Ray, Chaponda Mike, Kabuya Jean Bertin, Mulenga Modest

P12 Development of a quantitative HIV-1 and HIV-2 real time PCR (qRT-PCR) viral load assay

Moussa Thiam, Omar Janha, Alberta Davis, Alfred Amambua-Ngwa, Davis C. Nwakanma, Souleymane Mboup, Assan Jaye

P13 Differential effects of sex in a West African Cohort of HIV-1, HIV-2 and HIV-1/2 dual infected patients: Men are worse off

Sanne Jespersen, Bo Langhoff Hønge, Joakim Esbjörnsson, Candida Medina, David Da Silva TÉ, Faustino Gomes Correira, Alex Lund Laursen, Lars Østergaard, Andreas Andersen, Peter Aaby, Christian Erikstrup, Christian Wejse, for the Bissau HIV Cohort study group

P14 HIV-infected adolescents in transition from pediatric to adult HIV care in Dakar, Senegal: sample characteristics and immunological and virological profiles

Siry Dieye, Moussa Sarr, Haby Sy, Helene D Mbodj, Marianne Ndiaye, Amy Ndiaye, Seydi Moussa, Assan Jaye, Souleymane Mboup^100^

P15 Molecular characterization of vertically transmitted HIV-1 among children born to HIV-1 seropositive mothers in Northern Tanzania

Balthazar M. Nyombi, Elichilia R. Shao, Innocent B. Chilumba, Sikhulile Moyo, Simani Gaseitsiwe, Rosemary Musonda

P16 Breast-fed HIV-1 exposed infants play catch up. A preliminary report

Pam Datong, Bucky Inyang, Sophia Osawe, Abel Izang, Chundung Cole, Felicia Okolo, Bill Cameron, Kenneth Rosenthal, Clive Gray, Heather Jaspan, Alash’le Abimiku, the INFANT study team

P17 The frequency of N348I mutation in patient failing combination antiretroviral treatment In Botswana

Boitumelo Seraise, Kerstin Andrea-Marobela, Sikhulile Moyo, Rosemary Musonda, Joseph Makhema, Max Essex, Simani Gaseitsiwe

## A1 We are pleased to present peer reviewed forum proceedings of the 2^nd^ synchronicity forum of GHRI/CHVIfunded Canadian and African HIV prevention and vaccine teams

**Forum objectives**

- GHRI-funded capacity building and HIV prevention research teams presented highlights of achievements
- Teams discussed how to jointly build on achievements for sustainability
- Provided an opportunity for inter-team collaboration, synchronize best approach to capacity building, mentoring of new researchers and building leadership
- Provided opportunities for informal discussions and networking among the teams.
- Teams learnt about recent advances in the area of African regulatory and ethics review process
- The forum proceedings was a special supplement in an open-access journal was produced

**Forum partners**

- Canada-Africa Prevention Trials (CAPT) Network
- TanZamBo Project
- Kenya AIDS Vaccine Initiative (**KAVI**)
- Nigerian HIV/AIDS Prevention Platform
- African Development of AIDS Prevention Trials (**ADAPT2**)
- Canada-Sub Saharan Africa HIV/AIDS Network (**CanSSA**)
- The Benin HIV prevention preparatory Team
- The Free State HIV prevention Team (SA)
- West African Platform for HIV Intervention Research (**WAPHIR**)

**Funding and supporting partners**

The Afri-Can Forum-2 was made possible by support grant from Canada’s Global Health Research Initiative (GHRI), a partnership of Canadian government agencies dedicated to generating research that is relevant to health and health system decision-making in developing countries.

GHRI partnership is composed of the Canadian Institutes of Health Research (CIHR), Foreign Affairs, Trade and Development Canada (DFATD), and the International Development Research Centre (IDRC).

Another important supporting partner in this Forum was the Alliance Coordinating Office (ACO) of the Canadian HIV Vaccine Initiative (CHVI)

**Forum planning committee**

Assan Jaye (Chair) - MRC, The Gambia (*WAPHIR*)

Janan Dietrich (Co-Chair) - PHRU, WITS, South Africa (*CAPT*)

Tanya Merke Epp - ACO/CHVI, Manitoba, Canada

Mark Wainberg - Magill University, Canada (*TanZanBo*)

Alash'le Abimiku - University of Maryland (*Nigeria Platform*)

Marc Cohen -GHRI, Canada

Mark Brockman - Simon Frazer University, Canada (*CANSSA*)

Clive Gray (Co-Chair) - University of Cape Town, South Africa (*CAPT*)

Esme Lanktree - GHRI, Canada

Allan Ronald - University of Manitoba/ACO

Elizabeth Dunning - CIHR, Canada

Thumbi Ndung'u - UKZN, South Africa (*CANSSA*)

Rika Moorhouse - (*CAPT*)

Debbie Douglas - CHVI/ACO, Canada

Lilja Jónsdóttir - PHAC, Canada

Jennifer Gunning - CIHR, Canada

**Message from the chairs**

Participants were all welcomed to the second special synchronicity forum of the *Afri-Can* partnership (Canadian-African partnership on capacity building in HIV prevention and intervention research), supported by Canada’s Global Health Research Initiative (GHRI). The African teams in this partnership comprised of 9 research networks, which collaborated closely with Canadian researchers that have established excellence in HIV prevention research in African-owned health institutions, enabled locally relevant research, high quality training programs and strengthened capacity for HIV clinical trials.

This meeting took stock of our achievements, shared successes, fostered inter-team collaboration and discussed ways to build on our experiences. The meeting was timely when all of the GHRI-funded programs came to a close before the 1^st^ of December 2014; further posing a fundamental challenge on how to sustain what was built in the present global climate of dire scarce research funding. We needed not only to maintain our Institutional infrastructure and capacity but, expand our impact to fundamentally change the HIV health research landscape in Africa and maintain research excellence for generations to come.

This is a challenge that all of us including funders require giving utmost commitment if we have to make the HIV pandemic history. Everyone that participated in this Forum was committed to this challenge. We thank the participants for all their effort and participation in the event.

Sincerely,

Assan Jaye DVM, PhD.

Chair, Planning Committee

Janan Dietrich MA, PhD,

Co-Chair, Planning Committee

Clive Gray MSc, PhD

Co-Chair, Planning Committee

**Acknowledgement**

The forum organizers would like to thank:

• GHRI and its partners: (CIHR/CHVI, IDRC, DFATD) for funding this meeting;

• Alliance Coordinating Office (ACO) staff (Tanya Merke Epp and Nikki Calma) for the Secretarial, coordination and arrangement support.

• University of Witwatersrand, Johannesburg, South Africa for hosting the event and on-site Committee members (Janan Dietrich & Clive Gray) for all the hosting and logistic arrangements.

• Our eminent guest speakers, session Chairs, special guests and presenters.

• And all participants in the Forum.

## Oral presentations

### Theme 1: Behavioral and Social Approaches to Prevention – BeSo

#### O1 Voluntary medical male circumcision for prevention of heterosexual transmission of HIV in adult males in Soweto: What do indicators and incidence rate show?

##### Hillary Mukudu^1^, Janan Dietrich^1^, Neil Martinson^1^, Benn Sartorius^2^

###### ^1^Perinatal HIV Research Unit (PHRU), Chris Hani-Baragwanath Academic Hospital, Johannesburg, South Africa, ^2^Department of Public Health Medicine, School of Nursing and Public Health, University of KwaZulu Natal, Durban, South Africa

####### **Correspondence:** Hillary Mukudu (mukuduh@phru.co.za) – Perinatal HIV Research Unit (PHRU), Chris Hani-Baragwanath Academic Hospital, Johannesburg, South Africa

**Background:** The biomedical prevention of HIV transmission that medical male circumcision confers was confirmed by three clinical trial setting and then rolled out in VMMC programs in sub-Saharan Africa. Data assessing the effectiveness of this under programmatic conditions is not available. Concerns about possible risk compensation in circumcised males after circumcision have been raised.

**Methods:** A cohort of 233 HIV seronegative adult males aged 18-40 years seeking circumcision at a public hospital were followed up for a period ranging from 161 to 765 days after which the HIV serostatus rate was ascertained. We also compared the HIV behavioral risk factors before and after circumcision by calculating odds ratio (OR) with the 95 % confidence interval and p-value using the McNemar’s test. Logistic regression was used to determine the predictors of proxy HIV risk, before and after circumcision.

**Results and Discussions:** HIV incidence post circumcision 2.64 (95 % CI 0.54 - 4.75) per 100 person years. No evidence of risk compensation post circumcision. However, the participants were almost three times (OR 2.7 95%CI 1.34-5.69, p = 0.003) more likely to have vaginal sexual intercourse after circumcision, attributable to the fact that 21 % had either their sexual debut after circumcision or did not have sexual intercourse in the six months before circumcision but after. Post-circumcision there was evidence of adoption of positive HIV prevention behaviour because participants were 3.5 times (OR = 3.5 95 % CI 1.88-7.14, p = <0.0001) more likely to perceive themselves to be at risk of HIV and 58 % (OR = 0.42 95 % CI 0.16-1.01, p = 0.05) less likely to use alcohol with sex after circumcision. After circumcision participants in the 25-40 years age group were more than two and a half times (OR 2.60 95%CI 1.14-5.91 p = 0.023) more likely to be at risk of HIV acquisition than those in the 18-24 years age group.

**Conclusion:** These findings underscore the effectiveness of medical male circumcision in a program setting not only as a biomedical but also as a behaviour change intervention for prevention of HIV transmission and that there is no evidence of risk compensation after circumcision.

#### O2 Developing a peer-led community mobilization program for sex workers in Soweto: HIV risk and demographics

##### Jenny Coetzee^1,2^, Janan Dietrich^1^, Kgaugelo Mokgatswana^1^, Rachel Jewkes^2^, Glenda E Gray^1,2^

###### ^1^Perinatal HIV Research Unit, Chris Hani Baragwanath Hospital, Soweto, South Africa; ^2^Medical Research Council of South Africa, Cape Town, South Africa

####### **Correspondence:** Jenny Coetzee (coetzeej@phru.co.za) – Perinatal HIV Research Unit, Chris Hani Baragwanath Hospital, Soweto, South Africa

**Background:** Sex work (SW) is illegal in South Africa making access to health and legal services challenging. In 2014 the South African National Aids Council launched a strategy to improve SW access to services. Prior to September 2013, no SW programme existed in Soweto, a township on the outskirts of Johannesburg, despite SW activities in the more than 600 drinking establishments.

**Methods:** We aimed to work across Soweto collecting data to monitor the development of a peer-led SW project. SW peer educators (PEs)(n = 10) were trained to provide health talks, take field notes, demonstrate and distribute condoms and complete fieldwork forms during daily outreach, and to conduct HIV counselling and testing (HCT) as part of a wellness check offered through an existing HCT Centre (using non-SW lay counsellors and nurses). Data recorded included the number of unique SWs contacted, contextual dynamics, HIV results, CD4 count (laboratory), and referral for termination of pregnancy. Between October 2013 and December 2014, 341 SWs used the clinic services, 328 undertook HCT. In July 2014 we implemented a peer-led HCT strategy. Ethical approval was obtained.

**Results and Discussions:** The age range for SWs was 18-69 years (median 34). They were tavern and hostel based. Overall HIV positivity was 42 %, with outreach data suggesting that many SWs already knew their HIV positive status and were either on treatment or defaulting treatment. Service users preferred to undertake HCT through our peer-led strategy (90 %). Uptake of CD4 counts was poor (n = 63,46 %; CD4 range 37-2047, median 449) due to the non-SW nurse, delays in patient flow and result delivery. In 12 months we infiltrated 4/38 suburbs, with >3500 predominantly female SWs accessing outreach services.

**Conclusions:** HIV prevalence may be higher than 42 % given that outreach data suggests many SWs already know their status and thus refuse the clinic services offered by PE. Treatment and prevention adherence mechanisms need to be explored. Peer-led HCT to encourage HIV testing amongst SWs is a promising approach to SW programming in South Africa, although this could be scaled up to include point-of-care testing in addressing the poor uptake of CD4 counts. Understanding the broader needs of this population is critical to continued tailoring of key populations’ programmes.

#### O3 Salient beliefs about adherence: A qualitative survey conducted as part of the demonstration study on "treatment as prevention" (TasP) and "pre-exposure prophylaxis" (PrEP) among female sex workers (FSWS) in Cotonou, Benin

##### Marylène Dugas*,* Luc Béhanzin, Fernand A. Guédou, Marie-Pierre Gagnon, Michel Alary

###### Université Laval, Québec, Canada

####### **Correspondence:** Marylène Dugas (dugas11@hotmail.com) – Université Laval, Québec, Canada

**Background:** Among the strategies that have demonstrated good potential to reduce the spread of HIV among FSWs are the preventive methods TasP and PrEP. While these strategies have proven effective in randomized controlled trials, their applications in natural context is less convincing because, among other things, of therapeutic adherence. This presentation concerns the results of a qualitative survey conducted on the salient modal beliefs (Theory of Planned behavior) related to therapeutic adherence and conducted as part of a demonstration and feasibility study of TasP and PrEP strategies in Cotonou, Benin.

**Methods:** The qualitative study was conducted among three types of FSWs: 1- HIV Positive under antiretroviral (ARV) treatment; 2- HIV Positive not under ARVs (CD4 > 350); 3- seronegative. The topics investigated during the semi-structured interview were: 1- Advantages and disadvantages of treatment adherence; 2- Normative aspects of the adherence; 3- Control of therapeutic adherence. The method of Gagné and Godin (1999) was used for the evaluation and analysis.

**Results and Discussions:** A total of 31 FSWs participated in this study. Regaining or maintaining health and enjoy protection against HIV were the most cited advantages. The main disadvantages were: side effects, the daily intake and, for infected women, having to take the medication for life. On the normative aspects, the majority of FSWs have cited the health care workers and other FSWs as the ones who would be most in favor of adherence, except for other FSWs who would have experienced some problems or side effects with ARVs. Among the factors that would favor a good adhesion behavior, FSWs cited the efficient supply of ARVs and food availability. For the component impeding adherence: the side effects or lack of positive effects, the travels or the fear that the HIV status is revealed by the possession of the tablets.

**Conclusions:** The results highlighted the most important elements for the adhesion behavior of FSWs in Cotonou and allowed the creation of training tools for therapeutic adherence, which take into account these beliefs, and allowed the development of a questionnaire for the following evaluation of adherence behavior during TasP / PrEP demonstration study.

#### O4 Relative perception of risk as a driver of unsafe sexual practices among key populations: Cases of fisherfolk and women and their partners involved in multiple sexual partnerships in Uganda

##### Rwamahe Rutakumwa, Martin Mbonye, Thadeus Kiwanuka, Sarah Nakamanya, Richard Muhumuza, Winfred Nalukenge, Janet Seeley

###### Medical Research Council/UVRI Uganda Research Unit on AIDS, Uganda

####### **Correspondence:** Rwamahe Rutakumwa (Rutakumwa@mrcuganda.org) – Medical Research Council/UVRI Uganda Research Unit on AIDS, Uganda

**Background:** Fisherfolk and city-based women involved in multiple sexual partnerships in Uganda face an increased risk of HIV. However, the literature on how individuals make decisions about risk and sexual partners is limited. We investigate the social construction of risk in order to establish how individuals prioritise a given risk based on its perceived threat.

**Methods:** Over a seven month period ending in January 2014, a qualitative study was conducted among fisherfolk around Lake Victoria, and women at high risk of HIV infection and their male partners in Kampala, Uganda. We purposively sampled and held repeat life history in-depth interviews with 50 (20 male and 30 female) participants; approximately half of these were from each of the two study sites. Data were analysed thematically by identifying and linking patterns and themes.

**Results and Discussions:** Fishermen feared drowning, and immediately on their return from fishing, they said they often engage in sex usually with sex workers to ‘cool off’, suggesting that the threat of HIV was perceived as less significant than drowning, and that sex has therapeutic qualities. Partners of fishermen expressed a constant fear of catching HIV because their men were promiscuous. However, these women chose to stay with their men because the risk of poverty arising from leaving their men was a bigger threat to them and their children. Women engaged in commercial sex reported spousal violence and lack of basic necessities as key motivators of their decision to leave their spouses, which through the lack of alternatives led them into sex work. Misconceptions about the risk of condom use, such as the fear of a condom breaking and disappearing inside a woman, led to a preference for sex without a condom by some women.

**Conclusions:** An individual’s perception of risk is in some contexts contingent upon her/his sense of other risks that they encounter in their daily life. A risk where the consequence is perceived as being far away may be considered less significant than more immediate threats. Attention to context specific perceptions of contingency of risk should be paid in the design of interventions that promote safe sex.

#### O5 Exploring the acceptability of new biomedical HIV prevention technologies among MSM, adolescents and heterosexual adults in South Africa

##### Millicent Atujuna, Melissa Wallace, Ben Brown, Linda Gail Bekker, Peter A. Newman

###### Desmond Tutu Foundation, University of Cape Town, Cape Town, South Africa

####### **Correspondence:** Millicent Atujuna (Millicent.atujuna@hiv-research.org.za) – Desmond Tutu Foundation, University of Cape Town, Cape Town, South Africa

**Background:** Efforts to combat the transmission of HIV continue with numerous biomedical prevention trials reporting varying levels of partial efficacy. While these interventions offer promise for HIV prevention, their success will hinge on their acceptability, particularly across various social contexts. South Africa, hardest hit by the HIV epidemic, is of particular interest as it grapples with the outcomes of a complex interaction between issues of race, gender and migration created by apartheid, as they play out in a changing political economy. In light of this, we examine the acceptability of a Vaccine, Microbicides and oral PrEP in three target groups, namely adolescents (15-17 years), heterosexual adults and MSM.

**Methods:** We present qualitative data from populations recruited from two communities in Cape Town. We conducted 6 focus groups (FGs) (5-9 participants per group N = 36) and 12 in-depth interviews (IDIs). Of these, we purposively conducted 2 FGs and 5 IDIs with participants naïve to biomedical prevention trials and 4 FGs and 7 IDIs with participants who have participated in biomedical prevention trials. In addition, we included 8 interviews with healthcare providers. The interviews and focus groups were conducted in Xhosa and transcribed and translated into English. We followed a framework analysis approach as we analyzed the transcripts using Nvivo 10.

**Results:** Overall, participants projected a willingness to use the products in instances where condom use was inconsistent or non-existent, in cases of rape, and, in situations of blood contact. Similarly, participants imagined that sexual relationships amongst discordant couples were now a possibility. Data also showed that while acceptability and willingness to use these products were positively influenced by participant’s knowledge and awareness of the products, their conceptualization of product dosing regimens and the varying levels of efficacy produced inconsistent results. Finally, acceptability and willingness to use the products were negatively affected by people’s prevailing socio-economic circumstances as well as their perception of their cultural expectations defined within a traditional religious framework.

**Conclusion:** While biomedical HIV interventions are important, the role of the social context on the acceptability and possible use of these products must be considered in the implementation phase.

### Theme 2: HIV Immunology and Genetics – ImGen

#### O6 HIV-susceptible target cells in foreskins after voluntary medical male circumcision in South Africa

##### Rushil Harryparsad^1^, Abraham J. Olivier^1^, Heather B. Jaspan^1^, Douglas Wilson^2^, Janan Dietrich^3^, Neil Martinson^3^, Hillary Mukudu^3^, Nonhlanhla Mkhize^4^, Lynn Morris^4^, Gianguido Cianci^5^, Minh Dinh^5^, Thomas Hope^5^, Jo-Ann S. Passmore^1,6^, Clive M. Gray^1,6^

###### ^1^Institute of Infectious Diseases and Molecular Medicine, University of Cape Town, Cape Town, South Africa; ^2^Department of Medicine, Edendale Hospital, KwaZulu Natal, South Africa; ^3^Perinatal HIV Research Unit, Soweto, South Africa; ^4^National Institute for Communicable Diseases, Sandringham, South Africa; ^5^Division of Infectious Diseases, Department of Medicine, Northwestern University Feinberg School of Medicine, Chicago, Illinois, USA; ^6^National Health Laboratory Services, Cape Town, South Africa

####### **Correspondence:** Rushil Harryparsad (HRRRUS001@myuct.ac.za) – Institute of Infectious Diseases and Molecular Medicine, University of Cape Town, Cape Town, South Africa

**Background:** Medical Male Circumcision (MMC) reduces the risk of HIV acquisition by up to 60 %, confirmed in a number of large clinical trials throughout Africa. MMC has also been shown to reduce the prevalence of other sexually transmitted infections (STIs), which in turn may impact HIV acquisition. We hypothesized that the underlying mechanisms for this protection may be removal of potential target cells for HIV infection and altered levels of keratinisation in men after MMC.

**Methods:** In a longitudinal study involving 2 clinical sites and 150 participants within South Africa, we investigated CD4+ T cell frequencies by immunofluorescent imaging in a subset of 10 HIV negative individuals (14 – 24 years) undergoing elective MMC at Edendale Hospital in Kwa-Zulu Natal and at the Perinatal HIV Research Unit in Soweto, Johannesburg. We compared the levels of keratinisation between the inner and outer foreskin and assessed the impact of STIs (*C. trachomatis*, *N. gonorrhoea, M. genitalium*, *T. vaginalis, HSV-1 & 2*) on HIV target cell density in foreskin tissues. Tanner staging and testosterone levels were measured in all men included in the study.

**Results and Discussions:** Preliminary immunofluorescent staining for CD4, Ki67 and CD207 to identify proliferating immune cells and filaggrin for keratin layers showed elevated numbers of both CD4+ T and CD207+ Langerhans cells in the foreskin of men with STIs compared to those without an STI.

**Conclusions:** MMC may reduce the risk of HIV infection in this highly susceptible age group of men by removing the potential CD4+ HIV target cells present in foreskins of young uncircumcised men in South Africa. STI-induced inflammation and recruitment of immune cells to the foreskin, may be elevating the risk of HIV acquisition in uncircumcised men.

#### O7 HIV-1 proteins activate innate immune responses via TLR2 heterodimers

##### Bethany M. Henrick, Xiao-Dan Yao, Kenneth L. Rosenthal, the INFANT Study Team

###### McMaster Immunology Research Centre, Michael G. DeGroote Institute for Infectious Disease Research, Department of Pathology & Molecular Medicine, McMaster University, Hamilton, Ontario, Canada

####### **Correspondence:** Kenneth L. Rosenthal (rosenthl@mcmaster.ca) – McMaster Immunology Research Centre, Michael G. DeGroote Institute for Infectious Disease Research, Department of Pathology & Molecular Medicine, McMaster University, Hamilton, Ontario, Canada

**Background:** Chronic immune activation is a fundamental driver of HIV-1 infection, replication and pathogenesis. Currently, HIV-induced immune activation is believed to be primarily driven by translocation of bacterial products from the HIV-infected gut into the systemic circulation. However, our understanding of HIV-induced innate immune activation remains incomplete. Here, we hypothesized that HIV-1 structural proteins, which persist in infected tissues, may serve as pathogen-associated molecular patterns (PAMPs) that drive immune activation.

**Methods:** These studies made use of TZMbl cells stably transformed to express Toll-like receptor 2 (TLR2)and primary human T cells.

**Results and Discussions:** Our results demonstrate that significantly increased HIV-1 integration occurred in cells expressing TLR2 compared to cells lacking TLR2. Mechanistically, this appeared to be due to a TLR2-mediated increase in CCR5 expression. Importantly, HIV-1 structural proteins p17, gp41 and p24 were shown to act as viral PAMPs and were recognized by TLR2 and its heterodimers leading to significantly increased immune activation via NFkB signaling. Using co-immunoprecipitation and a cell membrane dot blot method, we demonstrated direct protein interactions between p17, p24 and gp41 with TLR2, while only p17 and gp41 bound to TLR1. TLR2/1 heterodimer recognized HIV-1 p17 and gp41 leading to immune activation, while p24 signaled through TLR2/6. These results were confirmed using TLR2/1 siRNA knockdown assays which ablated p17 and gp41-induced cytokine production and through studies of HEK293 cells expressing selected TLRs. Interestingly, in the absence of TLR6, p24 bound to TLR2 and blocked signaling by p17 and gp41. Thus, providing a novel mechanism by which HIV may manipulate innate sensing.

**Conclusions:** Our results show for the first time that HIV structural proteins can serve as PAMPs that activate innate responses via TLR2 and its heterodimers. These findings have important implications for our fundamental understanding of innate immune activaiton by HIV and also may provide insight into the design of novel HIV vaccines since HIV proteins that serve as PAMPs have auto-adjuvant activity.

#### O8 Characterization of an innate factor in human milk and mechanisms of action against HIV-1

##### Bethany M. Henrick^1^, Xiao-Dan Yao^1^, Anna G. Drannik^1^, Alash’le Abimiku^2^, Kenneth L. Rosenthal^1^, the INFANT Study Team

###### ^1^McMaster Immunology Research Centre, Michael G. DeGroote Institute for Infectious Disease Research, Department of Pathology & Molecular Medicine, McMaster University, Hamilton, Ontario, Canada; ^2^Institute of Human Virology, University of Maryland School of Medicine, Baltimore, Maryland, USA

####### **Correspondence:** Kenneth L. Rosenthal (rosenthl@mcmaster.ca) – McMaster Immunology Research Centre, Michael G. DeGroote Institute for Infectious Disease Research, Department of Pathology & Molecular Medicine, McMaster University, Hamilton, Ontario, Canada

**Background:** Previously we demonstrated that immunodepletion of soluble Toll-like receptor 2 (sTLR2) from human breast milk significantly increased HIV infection *in vitro*. The objectives of this study were to characterize sTLR2 levels in human milk from HIV-infected and uninfected women and identify its mechanisms of action against HIV-1.

**Methods:** This study was approved by the McMaster Research Ethics Board and the University of Maryland, Baltimore and Plateau State Specialist Hospital Nigeria Institutional Review Boards. All participants provided voluntary written informed consent. Historic breast milk samples from Nigeria included HIV-infected (n = 40) and uninfected (n = 15) samples, and HIV-uninfected breast milk samples from Hamilton, Ontario (n = 13) were blindly tested. ELISA assays were used to measure TLR2 and HIV-1 p24.

**Results and Discussions:** Breast milk from HIV-infected women had significantly elevated levels of sTLR2 compared to uninfected breast milk. sTLR2 levels correlated with HIV-1 p24 and IL-15 in milk, thus suggesting a local innate compensatory response in the HIV-infected breast. Next we demonstrated that mammary epithelial cells and macrophages produced significantly increased levels of sTLR2 following exposure to HIV-1 proteins, p17, p24 and gp41 or the TLR2 ligand, Pam_3_CSK_4_. Further, sTLR2 directly interacted with p17, p24 and gp41 as shown by co-immunoprecipitation. Importantly, sTLR2 significantly inhibited cell-free R5 HIV-1 infection, inflammation and NFκB activation. Mechanistically, binding of sTLR2 to HIV-1 proteins inhibited a TLR2-dependent increase in CCR5 expression, thus resulting in significantly reduced HIV-1 infection.

**Conclusions:** Our findings showed that sTLR2 significantly inhibited HIV-induced NFκB activation and inflammation in a dose-dependent manner; sTLR2 bound directly to HIV structural proteins; and sTLR2 inhibited TLR2-dependent, HIV-induced increases in CCR5 co-receptor expression. Together, these likely contribute to significant decreases in HIV MTCT.

#### O9 Secretor status and susceptibility to HIV infections among female sex workers in Nairobi, Kenya

##### Nadia Chanzu, Walter Mwanda, Julius Oyugi, Omu Anzala

###### KAVI Institute of Clinical Research, University of Nairobi, Nairobi, Kenya

####### **Correspondence:** Nadia Chanzu (nadiachanzu@gmail.com) – KAVI Institute of Clinical Research, University of Nairobi, Nairobi, Kenya

**Background:** Blood group antigens are expressed on red blood cells however; these antigens can also be expressed on some other cells particularly on the surface of epithelial cells and may be found in mucosal secretions. The gene known to determine the secretion of these blood group antigens is the *Secretor Fucosyltransferase 2* (*FUT2*) gene. In many human populations 80 % secrete ABO antigens (termed secretors) while 20 % do not (termed non-secretors). Furthermore, there are disease conditions that are associated with secretor status. It is against this background that this study was proposed: To investigate associations between mucosal blood group antigen expression profiles (secretor status), *Secretor FUT2* gene polymorphisms and susceptibility to HIV infection among female sex workers in Nairobi, Kenya.

**Methods:** This study recruited 280 female sex workers from the well-established Pumwani Majengo cohort aged 18 to 65 years of age. Blood typing was determined using standard serological techniques using monoclonal antibodies to the ABH, Rhesus (D) and Duffy (Fya, Fyb) blood group antigens. Secretor phenotyping was determined using lectins specific to blood group H antigen in both salivary and female genital tract samples. This was correlated to the HIV sero-status.

**Results and Discussions:** Saliva testing showed that 212 (76 %) study cases were secretors and 68 (24 %) non-secretors. Based on HIV screening, 92 (32.9 %) were HIV-1 infected and 188 (67.1 %) HIV-1 uninfected. There was a correlation between HIV infections and secretor phenotypes. The proportion of secretors was significantly higher among women with HIV infection (77/92 = 83.7 %) in comparison to HIV un-infected women (135/188 = 71.8 %) (p = 0.029). Furthermore, the incidence of HIV infection was significantly higher among blood group A secretors (p = 0.028) in comparison to O secretors, but not B and AB.

**Conclusions:** These findings suggest the non-secretor phenotype may confer a certain degree of protection against HIV infection, as there were higher HIV infection incidence rates among ABH secretors.

#### O10 Natural Killer cell recall responsiveness to Gag-HIV-1 peptides of HIV-1 exposed but uninfected subjects are associated with peripheral CXCR6+ NK cell subsets

##### Moustapha Mbow^1^, Sabelle Jallow^2^, Moussa Thiam^1^, Alberta Davis^2^, Assane Diouf^3^, Cheikh T. Ndour^3^, Moussa Seydi^3^, Tandakha N. Dieye^1^, Souleymane Mboup^1^, Martin Goodier^4^, Eleanor Rilley^4^, Assan Jaye^1,2^

###### ^1^Laboratory of Bacteriology and Virology of Aristide Le Dantec University Hospital, Dakar, Senegal; ^2^Medical Research Council (MRC) Unit, Serrekunda, The Gambia; ^3^Clinic of Infectious Disease of Fann University Hospital, Dakar, Senegal; ^4^Department of Immunology and Infection, London School of Hygiene & Tropical Medicine, London, UK

####### **Correspondence:** Moustapha Mbow (moustaphazero@yahoo.fr) – Laboratory of Bacteriology and Virology of Aristide Le Dantec University Hospital, Dakar, Senegal

**Background:** Despite that NK cells are traditionally defined as innate immune cells, increasing evidences suggest that NK cells contribute to antigen-specific secondary immune responses upon both specific and non-specific activation. Although it has been reported in models of virus infections that secondary virus-specific NK cell responses are restricted to CXCR6^+^ and/or Thy1^+^ NK cell subsets, the function and phenotype of human NK cells to protect against HIV infection or limit HIV transmission remains unclear. Here we investigated effector responses and putative “memory” phenotype of NK cell in HIV-1 infected adults and their HIV-exposed but uninfected partners as well as HIV-negative controls.

**Methodology:** We selected 48 HIV-1 positive individuals and their HIV-negative partners from a sero-discordant cohort in Dakar, Senegal and 28 HIV-sero-negative subjects as controls. PBMCs were stimulated for 18 h with whole HIV-1 clade A peptide pools of Reg (Tat, Rev, Vif, Vpu, and Vpr) and Gag, and Env 15-mers overlapping by 10 amino acids. We used multiparametric flow cytometry to analyze NK cells including the functional markers CD107a and IFN-γ and CXCR6. Differences between groups were assessed using the nonparametric Krushal-Wallis H and Mann-Whitney U tests and correlations performed using the Spearman Rho test.

**Results:** The proportion of NK cells producing IFN-γ in response to Gag peptides were significantly higher in exposed-uninfected partners (2.2 % [IQR:1.4-3.1]) compared to their HIV-1 positive partners (1.5 % [IQR:1.1-2.0], p = 0.013) and controls (0.8 % [IQR:0.7-1.0], p < 0.001). Moreover, CD107a-expresssing NK cells in response to Gag peptides were higher among HIV-exposed but uninfected partners (3.4 % [IQR:2.8-4.3]) than among controls (1.9 % [IQR:1.3-2.7], p < 0.001). Interestingly, the CXCR6 mean fluorescence intensity of NK cells was positively correlated with IFN-g-producing NK cells in HIV-exposed infected (r = 0.49 p = 0.001) and uninfected (r = 0.47; p = 0.05) subjects but this was not the case in controls CD107a (r = 0.05; p = 0.98).

**Conclusion:** These data suggest that exposure of NK cells to HIV antigens may prime specific recall functional response that would be important for protection against HIV. Despite evidences that antigen-specific IL-2 CD4^+^ T cells might be important for NK cell function, such responses might be mediated by the CXCR6+ (putative “memory”) phenotype.

#### O11 Profiles of resistance: Local innate mucosal immunity to HIV-1 in commercial sex workers

##### Xiao-Dan Yao^1^, RW. Omange^2^, Bethany M. Henrick^1^, Richard T. Lester^3^ Joshua Kimani^4^, T. Blake Ball^2^, Francis A. Plummer^2^, Kenneth L. Rosenthal^1^

###### ^1^McMaster Immunology Research Centre, Michael G. DeGroote Institute for Infectious Disease Research, Department of Pathology & Molecular Medicine, McMaster University, Hamilton, Ontario, Canada; ^2^Univeristy of Manitoba and Public Health Agency of Canada, Winnipeg, Manitoba, Canada; ^3^BCCDC, University of British Columbia, Vancouver, British Columbia, Canada; ^4^Department of Medical Microbiology, University of Nairobi, Nairobi, Kenya

####### **Correspondence:** Kenneth L. Rosenthal (rosenthl@mcmaster.ca) – McMaster Immunology Research Centre, Michael G. DeGroote Institute for Infectious Disease Research, Department of Pathology & Molecular Medicine, McMaster University, Hamilton, Ontario, Canada

**Background:** Highly HIV-1-exposed seronegative (HESN) cohorts of female commercial sex workers (CSWs) were first identified in Kenya. Given that natural resistance to infection is primarily mediated by innate immune mechanisms as well as many studies suggesting that HESN women have novel immune responses in the genital mucosa associated with protection, the main objective of this study was to determine whether expression and function of innate sensing and signaling pathways were altered locally in the genital mucosa of HIV-1 resistant women in a well-characterized cohort of Kenyan CSWs.

**Methods:** This study was guided by the Helsinki Declaration on ethical principles for medical research involving human subjects. All studies were approved by the University of Nairobi/Kenyatta National Hospital Ethical Review Board and University of Manitoba Health Research Ethics Board. All participants provided written informed consent for sample collection and analysis. Participants from the Pumwani CSWs cohort were enrolled in 2009 for this study and divided into three groups: HESN, HIV-negative (HIV-N), and HIV-positive (HIV-P), based on their HIV seroconversion status at the time of collection. Mucosal samples collected included cervicovaginal lavage (CVL), cervical mononuclear cells (CMCs) and cervical epithelial cells (CECs). RNA was extracted from CMC and CECs and quantitative reverse transcriptase PCR (qRT-PCR) to quantitate gene expression. Freshly isolated CMCs were stimulated to assess responses to pathogen-associated molecular patterns (PAMPs) in vitro.

**Results and Discussions:** Our results demonstrated that selected innate pattern recognition receptors (PRRs) were significantly reduced in expression in CMCs from HESN compared to HIV-N and HIV-P groups. Although baseline levels of secreted cytokines were reduced in CMCs of HESN, they were highly stimulated following exposure to ssRNA40 in vitro. Importantly, CECs from HESN also expressed reduced levels of PRRs, but Toll-like receptor 3 (TLR3) and TLR7, as well as NFκB and AP-1 were highly expressed and activated. Lastly, inflammatory cytokines IL-1β, IL-8, and RANTES were detected at lower levels in the CVL of HESN compared to HIV-N and HIV-P groups.

**Conclusions:** Our study reveals a local microenvironment of HIV resistance in the genital mucosa consisting of a finely controlled balance of basal immune quiescence with a focused and potent innate anti-viral response that may be critical to resistance of sexual transmission of HIV-1.

### Theme 3: Capacity Building for HIV Prevention and Vaccine Research – CapB

#### O12 Early antiretroviral therapy and pre-exposure prophylaxis for HIV prevention among female sex workers in Cotonou, Benin: A demonstration project

##### Luc Béhanzin^1,2^, Fernand A. Guédou^1,2^, Nassirou Geraldo^1^, Ella Goma Mastétsé^1^, Jerôme Charles Sossa^3^, Marcel Djimon Zannou^4,5^, Michel Alary^2,6,7^

###### ^1^Dispensaire IST, Centre de santé communal de Cotonou 1, Cotonou, Bénin; ^2^Centre de recherche du CHU de Québec, Québec, Canada; ^3^Programme national de lutte contre le Sida et les IST (PNLS-IST), Cotonou, Bénin; ^4^Faculté des sciences de la santé, Université d’Abomey-Calavi, Cotonou, Bénin; ^5^Centre national hospitalier universitaire HMK de Cotonou, Bénin; ^6^Département de médecine sociale et préventive, Université Laval, Québec, Canada; ^7^Institut national de santé publique du Québec, Québec, Canada

####### **Correspondence:** Luc Béhanzin (bphilus2013@gmail.com) – Dispensaire IST, Centre de santé communal de Cotonou 1, Cotonou, Bénin

**Background:** In Benin, female sex workers (FSWs) are at the heart of the HIV epidemic. Within a combination prevention framework, we are currently carrying out a demonstration project on treatment as prevention (TasP) among HIV-positive FSWs (they receive a first-line ART regimen as per the Benin guidelines) and pre-exposure prophylaxis (PrEP) with Truvada® among those HIV-negative. The study is conducted at Dispensaire IST, a STI clinic dedicated to FSWs in Cotonou. It aims to assess the feasibility and usefulness of integrating TasP and PrEP to the combination prevention package offered to FSWs in Benin.

**Methods:** After a community preparedness phase and the development of a specific education program on adherence, we are currently recruiting 100 HIV-infected FSWs for TasP and 250 HIV-negative FSWs for PrEP. The recruitment period will last for one year, followed by an additional one year of follow-up. The actual recruitment visit is preceded by a screening visit two weeks earlier in order to determine the HIV status and assess other eligibility criteria. Through Day 14 and quarterly follow-up visits, we closely monitor treatment adherence and changes in sexual behaviour, using various tools including biological markers. We report on the first two months of the clinical phase of the study.

**Results and Discussions:** From 18^th^ September to 17^th^ November 2014, we screened 84 FSWs and out of them, 41 were recruited in the PreP arm and 17 in the TasP arm, with recruitment rates among eligible FSWs estimated at 93 % and 100 %, respectively. To 17^th^ November, the retention rate at Day 14 was 100 %. The most common side effects reported in the PrEP arm were restlessness, polyphagy and headache; all were minor and resolved within 2 weeks following initiation of treatment. It is of interest to note that the cumulative incidence of pregnancy was 7 % between the screening visit and the Day 14 follow-up visit.

**Conclusions:** The recruitment rate is on target and FSWs show a great interest in the study. Its successful completion will allow a better delineation of the role of TasP and PrEP in HIV prevention packages aimed at FSWs in the African context.

#### O13 Building capacity for HIV prevention trials: Preliminary data from a Nigerian cohort of HIV exposed sero-negatives (HESN)

##### Sophia Osawe^1,2^, Evaezi Okpokoro^2^, Felicia Okolo^1^, Stephen Umaru^1^, Rebecca Abimiku^1^, Sam Audu^1^, Pam Datong^1^, Alash’le Abimiku^1,2,3^

###### ^1^Plateau State Human Virology Research Centre (PLASVIREC), Jos, Nigeria; ^2^Institute of Human Virology (IHVN), Abuja Nigeria; ^3^Institute of Human Virology, School of Medicine, University of Maryland (UMD), Baltimore, USA

####### **Correspondence:** Sophia Osawe (Sosawe@ihvnigeria.org) – Plateau State Human Virology Research Centre (PLASVIREC), Jos, Nigeria

**Background:** Despite the significant gains globally on the battle against HIV/AIDS; 3.5 million people are still living with HIV/AIDS by the end of 2013 with new HIV infections annually. In the last 5 years, a series of innovative prevention tools have been added to the arsenal to combat the epidemic with some success. However an effective HIV vaccine is still crucial to ending the epidemic. HIV exposed Sero-Negative (HESN) individuals have been identified as an important group for HIV prevention studies. This study builds the capacity for successfully conducting future clinical trials in a resource limited setting as ours.

**Methods:** A total of 536 heterosexual HESN adults were enrolled by December 2012 into a cohort in Jos, Nigeria. Study enrollees and their HIV positive partners were screened, confirmed HIV sero-discordant and enrolled. At follow up visits behavioral data were collected and medical examinations and lab testing performed. Research team was trained to assess eligibility based on laboratory test results. Trackers were able to contact volunteers to remind them about their visits and make sure of their willingness to continue in the study.

**Results and Discussions:** Out of 536 HESN volunteers 256(47.9 %) were female and 278 (52.1 %) male with a mean age of 37 ± 9 years. There were a total of 98 (18.0 %) HESN terminated from the study, after a follow up period of 3 years and 10 months. We recorded deaths of HESN from natural causes (5) and that of their HIV positive partners from AIDS related illnesses (22). Testing HIV positive partners showed that 1/3 had detectable viral loads with ranges of 20-2,186,199 copies/ml. Baseline male condom use showed that couples with F + M- were less likely to use condoms consistently. HIV incidence is 3.2 % per 100 person months.

**Conclusions:** One of our aims was to build capacity of a research site to conduct clinical trials and be able to enroll and retain volunteers in a study. Our team successfully collected data and retained a cohort of HESN. Overall, male condom use in the cohort remained low despite regular. Viral load results also indicate that HESN are still at risk of getting infected.

#### O14 Equipping healthcare professionals with skills required for the conduct of clinical trials in an effort to build capacity. Lessons learned

##### Jacquelyn Nyange, Joyce Olenja, Gaudensia Mutua, Walter Jaoko, Gloria Omosa-Manyonyi, Bashir Farah, Maureen Khaniri, Omu Anzala

###### KAVI –Institute of Clinical research, Nairobi, Kenya

####### **Correspondence:** Jacquelyn Nyange (JNyange@kaviuon.org) – KAVI –Institute of Clinical research, Nairobi, Kenya

**Background:** KAVI Institute of Clinical Research (KAVI-ICR) in collaboration with the Universities of Manitoba and Toronto received a grant from the Global Health Research Initiative (GHRI) through IDRC to conduct capacity building to establish KAVI-ICR as a centre of excellence for the training of healthcare professionals with the skills required for the conduct of clinical trials in the region.

**Methods:** Using networks of research collaborators/Institutions that KAVI-ICR has established over years, Good Clinical Practice training (GCP) needs among the clinical trial teams were identified. The research institutions approached were only expected to cover the cost of the venue and lunches for the training period. Using a KAVI-ICR experiential GCP approach developed over a long standing of Clinical research experience, training modules were developed which included a Good Clinical laboratory Practice( GCLP) and Good Participatory Practice( GPP) component. In order to address the issue sustainability of the training program beyond the duration of the IDRC grant, members of staff from within the site were identified and mentored to continue GCP trainings within their clinical sites and the region.

**Results and Discussions:** Between 2010 and 2014 a total of 418 clinical site staffs and the numbers increased with increased awareness of the training opportunity. The trained staff included physicians, Nurses, community health workers Laboratory technologists, IT/Data Clerks and Managers, Research assistants/secretaries/administrators, Principal and Sub investigators, Clinical trial/study coordinators, monitors and auditors working in Clinical sites from Kenya, Uganda, Tanzania, Rwanda and Botswana.

**Conclusions:** The face to face GCP training was highly appreciated based on the feedback received from the training evaluation.

#### O15 Educational technology to support active learning for HIV researchers and planners

##### Anne Cockcroft, Kendra Tonkin, Indu Girish, Puna Mhati, Ashley Cunningham, Neil Andersson

###### CIET Trust, PO Box 1240, Gaborone, Botswana

####### **Correspondence:** Anne Cockcroft (acockcroft@fastmail.fm) – CIET Trust, PO Box 1240, Gaborone, Botswana

**Background:** Recent educational literature stresses the importance of techniques to support active learning. Participants in training offered to HIV researchers and planners come with experience and need to integrate new learning into their professional settings.

**Methods:** A three week intensive face-to-face training course in Botswana for 22 HIV researchers and planners from 12 SADC countries covered foundations of epidemiology, basic analysis of health data, introduction to research, and reviews of published articles. We used android tablet devices and the open access Socrative software to support active learning. Socrative allows immediate responses to sets of questions – pre-designed or “off the cuff” – which can be teacher- or student-timed, and with different options for displaying the responses. Single answer, multiple response and narrative responses are possible. The participants accessed Socrative via a free app. Prior to teaching about different concepts, the participants used the tablets to respond to questions to indicate their existing understanding. During the sessions, they tested their understanding by responding to questions on the tablets that related the concepts to potential scenarios they might encounter in their work. We also used the tablets and software to seek the views of the participants about the training and its relevance to them.

**Results:** All 22 participants actively used the Socrative software, although they had never used such software before. Results from the initial question sets helped teachers to tailor their sessions according to the needs of the participants. The Socrative exercises largely replaced paper exercises, and were well-received. Most (18) participants found using Socrative a good experience and none found it bad. All considered it had helped their learning and 18 agreed it had helped them reflect on their own experience and knowledge. Positive aspects highlighted by participants included instant feedback, the “one to one” aspect, exercises in applying the concepts, and being able to test one’s learning several times.

**Conclusions:** The active learning approach worked well with the participants in the training and was effectively supported by the educational technology we used. We intend to build on this experience for future face to face and distance learning for similar groups.

#### O16 From Lake Kivu (Rwanda) and Lake Malawi (Tanzania) to the shores of Lake Victoria (Uganda): Strengthening laboratory capacity through Good Clinical Laboratory Practice training

##### Bashir Farah, Jackton Indangasi, Walter Jaoko, Gaudensia Mutua, Maureen Khaniri, Jacquelyn Nyange, Omu Anzala

###### University of Nairobi, Kenya

####### **Correspondence:** Bashir Farah (BFarah@kaviuon.org) – University of Nairobi, Kenya

**Background:** The provision of clinical care requires adequate access to quality laboratory support. The majority of significant illnesses require laboratory confirmation of the diagnosis, and laboratory monitoring of the patient after the diagnosis has been made. The capacity to plan and implement an effective laboratory system that meets the requirements of quality management systems depends on the availability of highly-trained and motivated personnel that are not only technically competent, but also possess strong leadership and managerial skills. Capacity strengthening is not only acquisition of modern equipment and reagents but also sustained commitment to training and quality.

**Methods:** KAVI-ICR was awarded a four-year grant by the Global Health Research Initiative of Canada (GHRI-Canada) to establish a center of excellence for the training of healthcare professionals in the conduct of clinical trials. KAVI-ICR developed, piloted and delivered a course on Good Clinical Laboratory Practice (GCLP) and trained laboratory staff from four countries in the East African region. The modules were grouped according to the 12 Quality System Essentials, together with their respective learning objectives. The laboratory staffs trained from the four countries were from research, public health and private laboratories.

**Results and Discussions:** A total of 886 laboratory staff from Kenya, Uganda, Tanzania and Rwanda were trained were trained from 2010 to 2014. 35 % were trained in Kenya, 30 % in Tanzania, 20 % in Uganda and 15 % in Rwanda. In addition Laboratory management staff in each of trained laboratories were trained and encouraged to implement a quality management system. This included development and implementation of quality and technical manuals, standard operating procedures (SOPs), and a document control system.

**Conclusions:** The ultimate goal of the training program was to improve the quality of laboratory services, achieve compliance with GCLP standards, gain ISO 15189 accreditation, and facilitate better clinical care and collaborative research. It was clear that laboratory staff were motivated to improve the quality of their testing and services when working toward a concrete endpoint such accreditation. Enhancing laboratory capacity through training is essential for generating reliable and accurate data from clinical research, especially in resource-constrained settings.

### Theme 4: HIV Treatment and Clinical Intervention – TxCln

#### O17 Rilpivirine and etravirine resistance mutations in HIV-1 subtype C infected patients on a non-nucleoside reverse transcriptase inhibitor-based combination antiretroviral therapy in Botswana

##### Thabo Diphoko, Simani Gaseitsiwe, Victoria Maiswe, Thato Iketleng, Dorcas Maruapula, Keabetswe Bedi, Sikhulile Moyo, Rosemary Musonda, Mark Wainberg, Joseph Makhema, Vladimir Novitsky, Richard Marlink, Max Essex

###### Botswana Harvard AIDS Institute Partnership, 1836 Northring Road, Princess Marina Hospital, Gaborone, Botswana

####### **Correspondence:** Thabo Diphoko (tdiphoko@bhp.org.bw) – Botswana Harvard AIDS Institute Partnership, 1836 Northring Road, Princess Marina Hospital, Gaborone, Botswana

**Background:** Rilpivirine (RPV) and Etravirine (ETR) are the recently approved second-generation non-nucleoside reverse transcriptase inhibitors (NNRTI) for HIV treatment. There is a substantial cross-resistance HIV mutation profile between first-generation NNRTI and the second-generation drugs. Prevalence of resistance-associated mutations (RAM) to RPV and ETR has not been documented in Botswana.

**Methods:** A total of 274 HIV-1 *pol* sequences from participants failing Nevirapine or Efavirenz-containing regimens were analyzed. The selected genotypes, in the form of FASTA files were uploaded into the Stanford University HIV drug resistance database for HIV-1 genotype resistance interpretation. Forty- two sequences were from an adult antiretroviral therapy (ART) study and 232 from a prevention of mother-to-child transmission (PMTCT) study in Botswana.

**Results and Discussions:** Prevalence of RPV and ETR RAM in the adult ART study (n = 42) were: V90I (16.7 %), A98G (7.1 %), K101E (26.2 %), V108I (7.1 %), E138A (23.8 %), E138K (2.4 %), E138G (4.8 %), V179D (4.8 %), V179I (9.5 %), Y181C (26.2 %), Y188L (2.4 %), G190A (19 %) and K238T (2.4 %). The most prevalent mutation was Y181C, which causes intermediate resistance to RPV and ETR. The PMTCT cohort (n = 232) most prevalent mutations were Y181C and E138A (both 8.2 %). The E138A mutation reduces HIV-1's susceptibility to both drugs. The K101E mutation prevalence was 26.2 % and 6.5 % for adult ART and PMTCT studies, respectively. This mutation is linked with low-level resistance to RPV and ETR, and also affects NNTRIs. In total 108/274 (39.4 %) of patients harbored RPV and ETR RAMs. A total of 42.9 % of patients in the adult ART study had 3 or more NNRTI mutations at virologic failure.

**Conclusions:** Although RPV and ETR are not yet used in Botswana, HIV-1 mutations conferring resistance to them have been identified at 39.4 % in studied participants. The levels of NNRTI cross-resistance increases with the increase in number of mutations a patient has, and this was high in the adult ART study. These findings are crucial in patients displaying such profiles in receipt of these second-generation drugs in the future hence calls for genotyping of patients with prior nevirapine or efavirenz exposure before prescription of RPV or ETR-containing cART.

#### O18 From home-based HIV testing to initiation of treatment: The AIDS Support Organization (TASO) Experience with Home-based HIV Counselling and Testing (HBHCT) among Adolescents in Uganda, 2005-2011

##### Stephen Okoboi^1^, Livingstone Ssali^1^, Sam Kalibala^2^, Josephine Birungi^1^, Aggrey Egessa^1^, Jonathan Wangisi^1^, Lyavala Joanne Okullu^1^, Celestin Bakanda^1^, Francis Obare^3^

###### ^1^The AIDS Support Organization (TASO), Kampala, Uganda; ^2^HIV core/population Council, Washinghton, DC, USA; ^3^HIV core/population Council, Nairobi, Kenya

####### **Correspondence:** Stephen Okoboi (Okobois@tasouganda.org) – The AIDS Support Organization (TASO), Kampala, Uganda

**Background:** This paper examines the cascade of HIV testing, support and treatment services in Uganda under the TASO HBHCT program and compares the patterns among adolescents aged 10-19 years with those of adults aged 20 years and above.

**Methodology:** Data included individuals who were counselled and tested for HIV at their homes through the TASO HBHCT program. Analysis entailed simple frequencies to determine the proportions of adolescents and adults that: (i) tested positive among those who received HBHCT from 2005 to 2011; (ii) were enrolled in care and support programs at TASO centres among those who tested positive during HBHCT; (iii) were determined to be eligible for ART among those who were enrolled in care and support programs at TASO centres; and (iv) were initiated on ART among those who were determined to be eligible.

**Results:** Between 2005 and 2011, TASO tested a total of 55,228 clients aged 10 years and above through the HBHCT program; 40 % were adolescents aged 10-19 years. The proportion of adolescents who tested positive under the program was consistently lower than that of adults across the years (between 2 % to 5 % compared to between 10 % and 14 % among adults). The proportion of HIV-positive adolescents that were enrolled in TASO centres more than tripled from 9 % in 2005 to 32 % in 2006 and steadily increased to 41 % in 2008. By contrast, the proportion of HIV-positive adults that were enrolled in TASO centres increased from 21 % in 2005 to 31 % in 2006 before levelling off at 33 % in 2007.The proportion of adolescents who were found to be eligible for ART more than doubled from 15 % in 2006 to 40 % in 2011 while the proportion of eligible adults increased from 16 % in 2006 to 36 % in 2007 but fluctuated between 30 % and 35 % thereafter. Among those who were eligible for ART between 2005 and 2011, 89 % of adolescents and 93 % of adults were initiated on ART at TASO.

**Conclusions:** The HBHCT program contributed to improved uptake of HIV services among adolescents aged 10-19 years who would otherwise not have accessed the services at all or in time.

#### O19 Feasibility study on using real time medication monitoring among HIV infected and Tuberculosis patients in Kilimanjaro, Tanzania

##### I. Marion Sumari-de Boer^1^, Hadija H. Semvua^1^, Jossy van den Boogaard^1^, Krisanta W. Kiwango^1^, Kennedy M. Ngowi^1^, Pythia T. Nieuwkerk^2^, Rob E. Aarnoutse^3^, Ireen Kiwelu^1^, Eva Muro^1^, Gibson S. Kibiki^1^

###### ^1^Kilimanjaro Clinical Research Institute, Moshi, Tanzania; ^2^University of Amsterdam, the Netherlands; ^3^Radboud University, Nijmegen, the Netherlands

####### **Correspondence:** I. Marion Sumari-de Boer (m.sumari@kcri.ac.tz) – Kilimanjaro Clinical Research Institute, Moshi, Tanzania

**Background:** HIV infected and tuberculosis (TB) patients have difficulties in reaching adequate levels of adherence (>95 %) to treatment. Many factors may influence adherence. One way to intervene is real time medication monitoring (RTMM). We conducted a pilot study on the use of RTMM in a resource-limited setting where factors such as network connectivity and electrical power stability play role.

**Methods:** We enrolled five HIV infected and five TB patients from Kilimanjaro region, Tanzania. They took their medication from an RTMM device for three months. The device sent a signal through the mobile network when it was opened (so-called medication event) to a database. In the database, we recorded the usual time of intake. When the device was not opened on time, patients received a reminder SMS. After three months, we interviewed patients on acceptability of RTMM and carried out quantitative and qualitative analyses of the data.

**Results and Discussions:** Six patients (60 %) reached adequate adherence, two HIV infected (40 %) and four TB patients (80 %). In total, there should have been 1104 medication events. Nine-hundred-twenty-two events (84 %) were on time. In 455 events (41 %), a reminder was sent, but this was not correct in 170 events (37 %). This means that the network was not stable enough to send the signal on time. The median number of battery charging was one time. Qualitative results showed that nine patients found the device helpful. All mentioned that it helped to remind them; nine mentioned it keeps medication safe and seven found it easy to carry the device. However, six patients reported that the size was too big. The SMS was considered fine by nine patients, but five patients recommended to also get an SMS before usual time of intake. Five patients experienced the network problem by receiving a reminder while they had already taken their medication. One patient suggested solar power to be provided to avoid charging problems.

**Conclusions:** Based on the analyses, the device is considered useful in the resource-limited setting Kilimanjaro. In this setting, optimisation of the device should consider issues related to network connectivity, power supply, and the size of the device.

#### O20 Deaths still among sero-discordant cohort in Nigeria despite Access to treatment

##### Ruth Datiri^1^, Grace Choji^1^, Sophia Osawe^1,2^, Evaezi Okpokoro^2^, Felicia Okolo^1^, Stephen Umaru^1^, Rebecca Abimiku^1^, Samuel Audu^1^, Pam Datong^1^, Alash’le Abimiku^1,2,3^

###### ^1^Plateau State Human Virology Research Centre (PLASVIREC), Jos, Nigeria; ^2^Institute of Human Virology (IHVN), Abuja, Nigeria; ^3^Institute of Human Virology, School of Medicine, University of Maryland (UMD), Baltimore, USA

####### **Correspondence:** Ruth Datiri (Ruth_datiri@yahoo.com) – Plateau State Human Virology Research Centre (PLASVIREC), Jos, Nigeria

**Background:** Low CD4 counts and high HIV viral load are known predictors of HIV transmission and mortality in HIV positive individuals. Since the introduction of ART in many resource limited countries like Nigeria, the number of deaths attributed to HIV infection have drastically reduced, however deaths that should have been averted due to the wide spread treatment programs are still occurring. We explored the reasons for partner deaths in a cohort of sero-discordant couples followed for 2 years.

**Methods:** A total of 536 volunteers were enrolled with their HIV positive partners and followed up for 2 years in Jos, Nigeria. Data on partner deaths were collected through a follow up period from November 2011 to August 2014. Death of partners was a criterion for couple termination and therefore exiting the study.

**Results and Discussion:** Among 536 enrollees and their HIV positive partners, we recorded a total of 23 deaths (4 %). Out of 23 deaths 10 (43.5 %) were females and 13 (56.5 %) were male with a mean age of 37 years old. Eleven (48 %) deceased HIV positive partners reported never using male condoms (OR 1.4). The average CD4 count and viral loads of the deceased HIV+ partners were 235 cells/μl and 257,445 copies/ml respectively despite being on ARTs for an average of 5 years. A majority of recorded deaths were due to AIDS related complications underscoring the need for better adherence to drugs.

**Conclusions:** Our study shows that sero-discordant couples in Nigeria and possibly in other African countries still experience loss of their HIV positive partners despite treatment programs. Our data indicates that more efforts need to be directed towards support and adherence counselling for partners in care and treatment programs in addition to drug resistance monitoring. Such efforts would also improve retention for HIV prevention studies including HIV vaccine trials. We found low use of condoms by couples due to reasons like procreation, religious belief and male partner refusal to use male condoms.

### Theme 5: HIV Vaccine Research and Vaccine Trials – Vac

#### O21 Therapeutic HIV-1 vaccine trials in Denmark and Guinea-Bissau

##### Fomsgaard A^1^, Karlsson I^1^, Jensen KJ^1^, Jensen SS^1^, Leo-Hansen C^1^, Jespersen S^2^, Da Silva Té D^3^, Rodrigues CM^3^, da Silva ZJ^4^, Janitzek CM^1^, Gerstoft J^5^, Kronborg G^5^, the WAPHIR Group^6^

###### ^1^Department of Virology, Statens Serum Institut, Copenhagen, Denmark; ^2^The Bandim Health Project, Bissau, Guinea-Bissau; ^3^Centro de Tratamiento Ambulatório, Hospital Nacional Simão Mendes, Bissau, Guinea-Bissau; ^4^National Laboratory, Bissau, Guinea-Bissau; ^5^Department of Infectious Diseases, Rigshospitalet, Copenhagen, Denmark; ^5^Department of Infectious Disease Hvidovre Hospital, Hvidovre, Denmark; ^6^Senagal

####### **Correspondence:** Fomsgaard A (afo@ssi.dk) – Department of Virology, Statens Serum Institut, Copenhagen, Denmark

**Background:** Therapeutic HIV vaccine is being pursued as part of a functional cure for HIV/AIDS. We designed a universal peptide vaccine (AFO18) matching the populations and HIV-1 strains in two distant geographic regions, Denmark, and Guinea-Bissau, West Africa. The vaccine represents 15 subdominant CTL epitopes restricted to 5 common HLA supertypes + 3 Th epitopes in the adjuvant CAF01.

**Methods:** Two single-blinded phase-I therapeutic HIV-1 vaccine trials are referred to, one in Denmark and one in Guinea-Bissau. Capacity to conduct clinical HIV vaccine trials in West Africa was built with WAPHIR during the study. Treatment-naïve HIV-1-infected individuals with high CD4 counts where immunized i.m. (week 0, 2, 4, 8) with AFO18. Placebo groups received saline.

**Results and Discussions:** In Denmark 11 individuals (10 vaccinees + 1 placebo), and in Guinea-Bissau 18 individuals (15 vaccinees + 3 placebo) completed all vaccinations and 6 months of follow-up. The vaccine was safe and well tolerated. No overall or sustained changes in viral-load or CD4^+^ T cell counts occurred; however, new T cell responses specific for one or more vaccine epitopes were induced (IC-FACS and/or ELISpot). Surprisingly, the immunogenicity of the T-cell vaccine as well as the pre-existing T-cell immunity to HIV-1 seem to be lower in this West African HIV-1 positive population as compared to the Danish population.

**Conclusions:** We show that it is possible to generate new T cell responses in treatment-naïve HIV-1-infected individuals using peptides in adjuvant and thereby redirect immunity to selected subdominant conserved CTL epitopes. We demonstrate that it is possible to design a broad vaccine that is immunogenic in two very different geographic regions. This broad T cell vaccine is an important proof-of-concept for further development preferentially using stronger adjuvant (CAF09) and during (early started) ART to minimize viral-load and restore immune function.

#### O22 Willingness to participate in a HIV vaccine Trial among HIV exposed sero-negative (HESN) persons in Jos, Nigeria

##### Evaezi Okpokoro^1^, Sophia Osawe^1, 2^, Ruth Daitiri^2^, Grace Choji^2^, Stephen Umaru^2^, Felicia Okolo^2^, Pam Datong^2^, Alash'le Abimiku^1,2,3^

###### ^1^Institute of Human Virology, Abuja Nigeria; ^2^Plateau State Human Virology Research Centre, Jos, Nigeria; ^3^Institute of Human Virology, School of Medicine, University of Maryland, Baltimore, USA

####### **Correspondence:** Evaezi Okpokoro (eokpokoro@ihvnigeria.org) – Institute of Human Virology, Abuja Nigeria

**Background:** Nigeria has the second highest burden of HIV infection following South Africa. The presence of subtype G virus in Nigeria offers a unique opportunity to examine the efficacy of an appropriate vaccine on a well characterized cohort of HIV exposed sero-negative (HESN) persons. In preparation for a future HIV vaccine trial, we document here the willingness of HESN persons in a discordant relationship residing in Jos Nigeria to participate in such a trial.

**Methods:** We developed a prospective cohort study in the format of mock clinical trial and followed up participants for 2 years. We provided prerequisite risk reduction counseling and HIV testing during the 10 follow-up visits over the 2 years follow-up and administered semi structured interviewer based questionnaire to assess willingness to participate in HIV vaccine trials among others. We analyzed correlated proportions at visits 1 and 10 using Mcnemars exact test.

**Results and Discussions:** We screened 660 persons and 534 were enrolled. We had a mean age 37 ± 9 years (19-65 years) and 48 % (256/534) being female HESN respondents. At enrollment, only about 14 % (72/534) had heard about HIV vaccine and this became 100 % at visit 10 (p value < 0.001). Also at enrollment, 99.2 % (530/534) were willing to participate in HIV vaccine trial and 97.4 % (520/534) would allow someone under their care to participate in such a trial. These became 100 % also as the study progressed to visit 10 (p value = 1.0 and 0.036 respectively).

**Conclusions:** HESN partners in a discordant relationship in Nigeria are willingness to participate in HIV vaccine trial. Thus funding a candidate vaccine among this group is crucial in sustaining this willingness.

#### O23 Clinical research volunteers’ perceptions and experiences of screening for enrolment at KAVI-Institute of Clinical Research, Kenya

##### Nyariki Emily, Olenja Joyce, Lorway R Robert, Anzala Anzala

###### University of Nairobi, Nairobi, Kenya

####### **Correspondence:** Nyariki Emily (nyarikiemily@yahoo.co.uk) – University of Nairobi, Nairobi, Kenya

**Background:** The goal of clinical research is to develop knowledge and advance medical treatments to improve human health outcomes. It involves recruitment of volunteers who must not only be willing to be enrolled and retained to participate in the studies to completion but also meet the eligibility criteria. At KAVI-Institute of Clinical Research, Kenya data from recruitment sites reveal that some eligible volunteers fail to turn up for enrollment. Studies have suggested that understanding participants’ perceptions and research experiences not only provides valuable measure of their ethical treatment but is critical for enhancing their overall research experiences and outcomes. In response, a study on volunteers’ perceptions and experiences of clinical research participation in Kenya was conducted among KAVI-ICR volunteers in 2014. This study explored volunteers’ perceptions and experiences at all stages of participation and potential impact on motivation and decision making.

**Methods:** A mixed method approach was applied. A survey questionnaire was administered to 166 volunteers from 6 selected KAVI-ICR studies. Out of 166 volunteers 44 were purposively selected for in-depth interviews and 4 narratives. In addition 6 peer educators, 8 trial staff were interviewed as key informants. Quantitative data was coded and analyzed using SPPS. Qualitative data was transcribed verbatim, thematic themes identified for code tree development, coded and entered into Atlas ti for analysis.

**Results and Discussions:** In this paper we share qualitative findings on volunteers’ perceptions of screening for eligibility in clinical research participation. Volunteers expressed mixed reactions about the screening process and outcomes. Overall the medical examination and being confirmed to be in good health was most appreciated as most volunteers had for the first time received free and comprehensive medical check-ups. They however, volunteers indicated having had fears about test outcomes for HIV and serious ailments prior to screening. Additionally concerns about blood and mucosal samples were raised and these varied across gender and sexual orientation.

**Conclusions:** Findings suggest that beyond willingness to participate, understanding and consenting, gender, sexual orientation and study type shapes volunteers’ perceptions and experiences with study requirements, procedures and processes; that ultimately influence motivation to participate in clinical research.

#### O24 Gut microbiome, HIV-exposure, and vaccine responses in South African infants

##### Katie Viljoen, Jerome Wendoh, Elvis Kidzeru, Ulas Karaoz, Eoin Brodie, Gerrit Botha, Nicola Mulder, Clive Gray, William Cameron, Alain Stintzi, Heather Jaspan, for the INFANT study team

###### Division of Immunology, Institute of Infectious Disease and Molecular Medicine, University of Cape Town, Cape Town, South Africa

####### **Correspondence:** Heather Jaspan (hbjaspan@gmail.com) – Division of Immunology, Institute of Infectious Disease and Molecular Medicine, University of Cape Town, Cape Town, South Africa

**Background:** The gut microbiome is crucial for mucosal and systemic immune development. In mice, certain bacteria are required for induction of Treg and Th17 cell development in the gut. Likewise, gut microbiota enhance immune responses to influenza vaccination. HIV-infected women have altered vaginal and gut microbiome, and HIV-exposed infants (HEU) and their mothers receive antibiotics for PCP prophylaxis, therefore HEU may have altered gut microbiota. HEU have increased morbidity and mortality than HIV-unexposed (HU) infants, and respond poorly to certain infant vaccinations. We hypothesized that the etiology of this relative immune deficiency is mediated by gut dysbiosis.

**Methods:** HEU and HU infants were recruited from informal settlements of Cape Town. Stool DNA was extracted using MoBio PowerFecal DNA kit and 454 or Illumina sequencing was performed. Data was preprocessed using QIIME and UPARSE and imported into R for further analyses using the R package phyloseq. Differential abundance testing was performed at OTU level using the R metagenomeSeq package. Whole blood was incubated with BCG and proliferation and cytokine expression measured using multiparameter flow cytometry.

**Results and Discussions:** We found substantial differences in bacterial diversity between HEU and HU infants by Shannon index. Moreover, at all taxonomic levels, there were differences between the groups via PCoA analysis. Several OTUs of the phylum Firmicutes were differentially abundant between HIV exposed and HIV-unexposed infants, three of which were of the genus Veillonella. In HU infants, several key species were significantly correlated with both proliferative and cytokine responses to BCG. For example, at 6 weeks of age, significantly decreased abundance of *Bacteroides* spp. and in particular *B. fragilis* were present in infants with high CD4 + IL-2, CD8 + ki67+, and CD8 + IL-17+ responses at week 6.

**Conclusions:** Gut microbial makeup could explain the immunological differences between HU and HEU infants. Although the differences persisted after adjustment for feeding status, future work will focus entirely on breastfed HEU infants in the INFANT study.

#### O25 Analysis of HIV pol diversity in the concentrated HIV epidemic in Saskatchewan

##### Paul N. Levett, David Alexander, Naveed Gulzar, Prabvir S. Grewal, Art F.Y. Poon, Zabrina Brumme, P. Richard Harrigan, James I. Brooks, Paul A. Sandstrom, Stryker Calvez, Stephen E. Sanche, Jamie K. Scott

###### Dept. of Molecular Biology & Biochemistry, Faculty of Science and Faculty of Health Sciences Simon Fraser University, University Drive Burnaby, Unceded Coast Salish Territories, Canada

####### **Correspondence:** Jamie K. Scott (jkscott@sfu.ca) – Dept. of Molecular Biology & Biochemistry, Faculty of Science and Faculty of Health Sciences Simon Fraser University, University Drive Burnaby, Unceded Coast Salish Territories, Canada

**Background:** My laboratory is interested in testing the hypothesis that concentrated HIV epidemics, in which HIVs of limited genetic diversity are circulating, are valuable populations for HIV vaccine development and testing. This hypothesis relates to the goals of the Botswana-Canada AIDS Vaccine Discovery Partnership; however challenges have arisen in setting up the latter study and sample collection is only now beginning. Thus, in the interim, my lab has sought to study HIV genetic diversity in a concentrated epidemic here in Canada; that is, the HIV epidemic in Saskatchewan.

**Methods:** Plasma samples collected from patients entering clinics in Saskatchewan were sent to the Public Health Agency of Canada (PHAC) for routine HIV *pol* sequencing. Residual plasma samples were shipped to Simon Fraser University, and the same pol region, and also the V3 region of the HIV envelope gene (env) were sequenced.

**Results and Discussions:** Analysis by collaborator, Paul Levett (SK Disease Control Laboratory), of *pol* sequences from newly diagnosed infections obtained by PHAC between 2004 and 2012 indicate that HIV is somewhat diverse, but also shows differential clustering in two urban regions, and with patterns that have been stable over time. Our phylogenetic analyses of the HIV env V3 region showed similar characteristics. We have begun cloning full-length *env* from plasma samples, and collaborators at University of Saskatchewan are setting up to collect blood from ~250 HIV+ participants over the coming 9 months. We will be testing the hypothesis that HIV+ individuals who harbor viruses with closely related *envs* will produce antibodies that will “cross-neutralize” each other’s viruses. A positive result would support further testing of these dominating *envs* as vaccine candidates.

**Conclusions:** The impact of the proposed study is two-fold. First, while a multi-envelope vaccine will not target all circulating strains in Saskatchewan, or those of a comparable concentrated epidemic, it may provide protection against Saskatchewan’s dominant circulating strains (proving vaccine efficacy). Second, demonstration of antibody-mediated protection in humans would finally bring unambiguous positive results to the HIV-vaccine research effort.

## Poster presentations

### Theme 1: Behavioral and Social approaches to prevention – BeSo

#### P1 Evaluating a HIV vaccine research community engagement programme at two HIV prevention research centres in the Western Cape

##### Leslie Swartz, Ashraf Kagee, Anthea Lesch, Zuhayr Kafaar, Anneliese De Wet

###### Stellenbosch University, Stellenbosch, South Africa

####### **Correspondence:** Anneliese De Wet (anneliesedewet@sun.ac.za) – Stellenbosch University, Cape Town, South Africa

**Background:** In developing an HIV vaccine, the clinical trial context poses some intrinsic and extrinsic challenges, which need to be taken into account when evaluating community engagement processes. The intrinsic challenges are recruiting and retaining participants for the duration of trial and implementing the highest quality standards in trial protocol while, at the same time, leaving the community better off. The extrinsic challenges are dovetailing clinical trial efforts with other HIV risk reduction strategies, addressing social and contextual issues, such as poverty, unemployment and education, in communities with individuals at high risk of HIV infection, as well as respecting human rights and dealing with ethical dilemmas in the process. These challenges emphasis the importance of the involvement of the community in HIV vaccine trials (HIVVTs). Communities are, however, complex. Efforts to develop a safe, efficacious HIV vaccine must be based on collaboration and partnerships among all stakeholders involved. The aims of the study are to describe the context of community participation with regard to HIV vaccine trial participation and to evaluate current activities and approaches to community participation and knowledge dissemination.

**Methods:** The research is being conducted in two phases. The study is being conducted at the Masiphumelele HIVVT site and the Men’s Research division of the Desmond Tutu HIV Foundation (DTHF). The participants consist of community engagement staff and community members. Interviews and focus group discussions are being conducted. The interviews and focus group discussions are audio-recorded and transcribed and the data obtained analysed with the use of thematic analysis as described by Braun and Clarke (2006).

**Results and Discussions:** The data are being collected and analysed at present.

**Conclusions:** This research aims to contribute to the knowledge on developing contextually relevant and culturally sensitive community engagement activities that align with existing processes in two local communities. This community participation project is funded by CHVI.

#### P2 Validating HIV acquisition risk score using a cohort HIV exposed sero-negative persons in a discordant relationship in Jos, Nigeria, West Africa

##### Evaezi Okpokoro^1^, Sophia Osawe^1, 2^, Ruth Daitiri^2^, Grace Choji^2^, Stephen Umaru^2^, Felicia Okolo^2^, Pam Datong^2^, Alash'le Abimiku^1, 2, 3^

###### ^1^Institute of Human Virology, Abuja Nigeria; ^2^Plateau State Human Virology Research Centre, Jos, Nigeria; ^3^Institute of Human Virology, School of Medicine, University of Maryland, Baltimore, USA

####### **Correspondence:** Evaezi Okpokoro (eokpokoro@ihvnigeria.org) – Institute of Human Virology, Abuja Nigeria

**Background:** HIV sero-discordancy is reported as a preventable source of new infections. The provision and impact of biomedical prevention programs such as the pre-exposure prophylaxis can be optimized when high risk HIV exposed sero-negative (HESN) persons are targeted. Toward this end, a risk scoring tool was developed and published by Kahle et al (2013) using data from South and East African countries. We validate this empirical tool using a cohort of HESN residing in Jos, Nigeria, West Africa.

**Methods:** The original study enrolled and followed up 500 HESN for 2 years based on define eligibility criteria such as: established HIV sero-discordance with at least 3 months in the relationship; above 18 years etc. Following ethical approval and informed consent, we administered standardized questionnaires on risk behavior, medical history and performed safety lab test. We also obtained medical history and performed CD4 and viral load test on HIV+ partners of this cohort. To apply the risk score to this cohort, we modified the tool to exclude “number of children” and “male HIV 1 uninfected partner uncircumcised” as used in the original risk scoring tool developed by Kahle et al (2013) as circumcision is culturally practiced in over 95 % of Nigerian males.

**Results and Discussions:** Seventy four percentage of 534 eligible HESN enrolled had a risk score of 3 (i.e. low risk score) and an overall HIV sero-incidence of 4.2 per 10000 persons month. However HESN who sero-converted were 4 times more likely to have a risk score of at least 4 when compared with HESN who remained HIV negative (RR 3.83, fishers exact p = 0.005).

**Conclusions:** Our data validates the sensitivity of the risk-scoring tool in our cohort of Nigerian HESN despite the exclusion of male circumcision and children in our modified risk score. We recommend the adaptability of the risk score tool based on prevailing risk factors in different communities especially with limited resources and the need to target the most at risk first with available tested prevention programs.

#### P3 Bridging the gap between adults and adolescents and youth adults (AYA) – Employing a youth-centred approach to investigate HIV risk among AYA in Soweto and Durban, South Africa

##### Janan Dietrich^1^, Tricia Smith^2^, Laura Cotton^2^, Stefanie Hornschuh^1^, Martin van der Watt^1^ Cari L. Miller^2^, Glenda Gray^1^, Jenni Smit^3^, Manjeetha Jaggernath^4^, Thumbi Ndung’u^4^, Mark Brockman^2^, Angela Kaida^2^, on behalf of the AYAZAZI study teams

###### ^1^Bio-Behavioural Research, Perinatal HIV Research Unit, Soweto, Johannesburg, South Africa; ^2^Faculty of Health Sciences, Simon Fraser University, British Columbia, Canada; ^3^MatCH Research, University of the Witwatersrand, Durban, South Africa; ^4^HIV Pathogenesis Programme, University of KwaZulu-Natal, Durban, South Africa

####### **Correspondence:** Tricia Smith (Tricia2679@yahoo.com) – Faculty of Health Sciences, Simon Fraser University, British Columbia, Canada

**Background:** Adolescents and young adults (AYA) (10-24 years) are significantly at risk of HIV acquisition across Southern Africa. The continued burden of HIV transmission among AYA reflects poor prioritization of youth-focused and engaged research yielding an inadequate understanding of intersecting factors necessary to implement accessible and effective HIV prevention programs. Use of youth-centred approaches within HIV research and programming presents a critical opportunity to engage youth, build capacity, and support youth leadership in the HIV response.

**Methods:***AYAZAZI* (‘Knowing Themselves’ in Zulu) is a youth-centred, inter-disciplinary cohort study aiming to link socio-behavioural, structural, clinical, and biomedical data to understand HIV acquisition risk among AYA aged 16-24 years living in two study sites: Soweto and Durban, South Africa. We aim to enroll a cohort of 400 AYA (HIV-negative or HIV status unknown). AYAZAZI uses a youth-centred approach to engage and retain AYA throughout the research process. A Soweto-based Adolescent Community Advisory Board and youth engagement committee provides overall study guidance and oversight. At time of abstract submission, AYAZAZI Soweto had enrolled 28 males and 35 females. Preparations are underway to enroll participants in Durban in June 2015.

**Youth Engagement Activities:** We engaged the expertise and lived experiences of community members in the development of the survey and clinical protocol to ensure youth-appropriate language and priorities. AYA research assistants from Soweto were hired and trained to support recruitment and to administer surveys. AYA research assistants from Durban will likewise be hired and trained to do the same. We engaged with several youth-focused community partners to conduct outreach and raise awareness of AYAZAZI.

**Discussion:** AYA research assistants and their adult allies will receive ongoing training to support skill development and participants will have access to regular knowledge exchange forums to support youth leadership around HIV knowledge. Youth-centred, inter-disciplinary approaches require time and patience and the commitment of both young and older staff. Such research collaborations, however, that engage AYA and adult allies, are needed to better understand the realities of AYA’s and to build local capacity required to prevent HIV infection and reverse the high HIV risk environment among youth in South Africa.

#### P4 Neighbours to sex workers: A key population that has been ignored

##### Maureen Akolo^1^, Dr Joshua Kimani^2^, Prof Larry Gelmon^2^, Michael Chitwa^1^, Dr Justus Osero^3^

###### ^1^Partners in Health and Development in Africa, Nairobi, Kenya; ^2^University of Nairobi/ University of Manitoba-Nairobi Kenya; ^3^Kenyatta University-Nairobi, Kenya

####### **Correspondence:** Maureen Akolo (Molly_akolo@yahoo.com) – Partners in Health and Development in Africa, Nairobi, Kenya

**Background:** University of Manitoba/ Nairobi is an HIV/STI research center that has been in existence since mid 80,s. It has been focusing on STI/HIV research among sex workers and linkages to general population. Recently a clinical trial based on Inducing Immune Quiescence as a new avenue to prevent HIV infection was carried among HIV low risk childbearing age women sampled from two communities that are highly populated with sex workers (neighbors to sex workers) and the community aspect was analyzed.

**Methods:** The HIV Inducing Immune Quiescence (IIQ) clinical trial pre screened 158 women (neighbors to sex workers) within child bearing age in a period of one month from Majengo and Ngomongo slums in Nairobi. Exclusion criteria included HIV/STI positive, pregnancy, lactation, under age and menopause. The ones who met inclusion criteria were followed for 3 months and HIV/STI tests carried out monthly.

**Results and Discussions:** Out of the 158 study participants 9 (5.6 %) tested HIV positive during pre screening stage, 105 qualified for the study. None of the 105 sero-converted during the study period.17 (11 %) tested positive for STI. 98 % reported lack of condom use, the rest reported inconsistent condom use. One percent reported having two sex partners, while 96 % were married and none of them used condoms.

**Conclusions:** The HIV low risk women living within sex workers populated slums have high rates of STI which increases the risk of HIV acquisition and transmission. It is a group that should be followed keenly and treated as a key population. Further research should be done on this women sex partners/husband to ascertain if they are the regular partners to sex workers due to proximity and the rate of HIV/STI among their wives.

#### P5 Young women’s access to structural support programmes in a district of Botswana

##### Anne Cockcroft, Nobantu Marokoane, Leagajang Kgakole, Boikhutso Maswabi, Neo Mpofu, Umaira Ansari, Neil Andersson

###### CIET Trust, Gaberone, Botswana

####### **Correspondence:** Anne Cockcroft (acockcroft@fastmail.fm) – CIET Trust, Gaberone, Botswana

**Background:** A cluster randomized controlled trial in Botswana is testing the impact on HIV rates in young women of an intervention including making existing government structural support programmes more accessible to them. In the first intervention district we conducted an engagement survey of young women.

Methods: Trained female fieldworkers visited all households and interviewed women aged 15-29 years. They asked about awareness of programmes and experience when applying for support, and shared information about programmes via video clips and leaflets.

**Results:** Among 9,396 households visited, 1,838 had someone home and included at least one female member aged 15-29 years. Among 2,841 young women identified in these households, 1,493 were available and interviewed (80 refused). Some 15 % of the young women had been students throughout the last 12 months, 2 % had been in full time employment throughout, and 40 % had been unemployed throughout.

Most (81 %) had had sex. In the last 12 months, 16 % of these had sex with more than one partner, 16 % had a partner more than 10 years older, and 14 % received money or gifts for sex.

Awareness of 11 structural support programmes, was variable; for five of the programmes less than half of the young women had heard of them. Many did not apply even though they were aware of programmes, and others applied unsuccessfully. Some 17 % had benefitted from Ipelegeng (short term employment at minimum wage), 2.5 % from Back to School, 2.3 % from a youth national service programme; rates for all others were less than 1 %. Most thought the programmes *could* be useful for young women, citing making money and improving livelihoods, increasing financial independence, and building skills and confidence. Problems included length and complexity of the application process, unhelpful officers, inaccessible offices, and lack of support or advice about projects. Almost all expressed interest in workshops to help them use the programmes. Efforts are underway to integrate programmes and improve logistic support for officers.

**Conclusion:** Vulnerable young women (not in education or employment) are not accessing structural support programmes but are interested to do so. Implementation research will follow their use and experience of programmes.

#### P6 Voices for action from peri-urban Ugandan students, teachers and parents on HIV/STI prevention: Qualitative research results

##### Nakinobe Elizabeth^1^, Miiro George Mukalazi^1^, Zalwango Flavia^2^, Nakiyingi-Miiro Jessica^2^, Kaleebu Potiano^1,2^

###### ^1^Uganda Virus Research Institute Entebbe, Entebbe, Uganda; ^2^MRC/UVRI Uganda Research Unit on AIDS Entebbe, Entebbe, Uganda

####### **Correspondence:** Nakinobe Elizabeth (kinobelisa@yahoo.com) – Uganda Virus Research Institute Entebbe, Entebbe, Uganda

**Background:** Early sex and risky behavior may result into new HIV infections and other consequences, which can be prevented and controlled through improved access to adolescent-friendly services. The Adolescent-Centered Engagement and Research for Empowerment (ACERE) is a school-driven multi-level intervention project with in-built operational research (https://www.facebook.com/groups/627284160648195/). It aims at reducing Sexually Transmitted Infections (STIs) and HIV through engaging: students, teachers, parents/guardians, municipal, education, health and law-enforcing authorities; health facilities (to offer a continuum of HIV prevention and care services), with researchers from UVRI. We investigated the challenges and opportunities for students to access adolescent-friendly services.

**Methods:** In 2013, an Education Officer randomly selected seven secondary schools in Entebbe Municipality, Uganda to participate in mixed research methods including Focus Group Discussions (FGDs) and survey. We conducted 20 gender-specific FGDs (6 for girls, 6 for boys, 4 among female teachers and 4 in male teachers), using a topic guide and trained facilitators. Each FGD comprised of 10 participants, with adolescents aged 12-19 years. Random participant selection and informed consent were conducted. All FGDs were audio-recorded, transcribed, and stored using Nvivo 8 software.

**Results and Discussions:** Students reported consequences of pornography, early sex, STIs, absenteeism, poor class performance, unplanned pregnancies and worrying school drop-out, coupled with illicit drug abuse and alcohol. They frequently mentioned: lack of sexual education, co-curricular activities and tailored health services; judgmental adult health workers; poor parental support and behavioral modelling. Most teachers reported their limited knowledge and skills to deliver sexual and life-skills education, yet parents had negligently left this role to them. However, parents were not comfortable with teachers talking to their children about sexuality and reproductive health. The survey revealed that children, whose parents never supported/encouraged them, were more likely (58 %) than those supported/encouraged (41 %) to have early sexual debut (p = 0.02). Children whose parents never listen to them were more likely (56 %) than those whose parents listen (39 %) to have early sexual debut (p = 0.004).

**Conclusions:** Tailor-made sex education material, youth peer-counsellors, social media skills, , improved parental communication and support, and co-curricular activities, enabling policies/laws on drugs and alcohol are urgently needed to overcome the challenges encountered.

#### P7 Engaging Social Media as an education tool on the fly: The use of Facebook for HIV and Ebola prevention and awareness amongst adolescents in Uganda

##### John Ross Semwanga^1^, Emily Nyanzi^1^, Saidat Namuli Musoke^1^, Elizabeth Nakinobe^1^, George Miiro^1^, Edward Katongole Mbidde^1^, Tom Lutalo^1,3^, Pontiano Kaleebu^1,2^

###### ^1^Uganda Virus Research Institute, Entebbe, Uganda; ^2^MRC/UVRI Uganda Research Unit on AIDS, Entebbe, Uganda; ^3^Rakai Health Sciences program, Entebbe, Uganda

####### **Correspondence:** John Ross Semwanga (jssemwanga@uvri.go.ug) – Uganda Virus Research Institute, Entebbe, Uganda

**Background:** The Uganda Virus Research Institute (UVRI) is mandated by the Ministry of Health to conduct scientific investigations on viral and other communicable diseases so as to contribute to knowledge, policy and practice. The Institute reconstructed its website in September 2013 when Facebook/Twitter links were added for interactive research dissemination and knowledge sharing purposes.

**Methods:** With funding from the Adolescent Centered Engagement and Research for Empowerment (ACERE) study in Entebbe, outreach activities for adolescents to pose and record questions on HIV, STIs, Medical Male Circumcision, alcohol and drug abuse were conducted. UVRI recruited a youth counsellor who received these questions and provided real time researched answers and also coordinated discussions on the Facebook page.

**Results:** By November 2014 the UVRI Facebook page clocked to 1000 users. Questions and topics evolved around adolescent attitudes towards pregnancies in the era of AIDS, men’s fear of responsibilities, whether washing after sex prevents HIV infection, proper use of condoms, UVRI experience with the Ebola outbreaks, differences between Ebola and Marburg and the dynamics of HIV, Ebola and Marburg infections.

The discussions tried to provide simple responses to those that visited the website. In the majority of cases users appreciated the simplicity of the responses and were satisfied with the explanations. The interaction happened throughout the working hours and the counselor tried to respond outside office hours.

**Conclusions:** Social media if properly managed can be a great tool in communicating research findings. It can be used for real time knowledge translation targeting interested users. There has been a great turn up, feedback and interactions from the rest of the world from these Medias, driving more traffic to the UVRI website and reached more audiences than thought. There is need to recruit and train social media managers in research communication and to develop guidelines on how to properly manage social media, generate more content and attract more followers.

### Theme 2: HIV Immunology and Genetics – ImGen

#### P8 Circulating HIV-1 subtypes among sexual minority populations in Zambia

##### Ray Handema, Graham P. Chianzu

###### Tropical Diseases Research Centre, Ndola, Zambia

####### **Correspondence:** Ray Handema (handemar@tdrc.org.za) – Tropical Diseases Research Centre, Ndola, Zambia

**Background:** Despite some significant decreases in the HIV prevalence in the general population, not much is known about the sexual minorities in Zambia. Currently, overall HIV prevalence in the general population in Zambia is at 12 %. HIV-1 subtype C is predominant. The HIV epidemic in Zambia has mainly been known to be confined to the heterosexual populations. Though denied, sexual minorities, i.e. lesbians, gays, bisexuals and transgender exist in Africa. LGBT persons in Zambia face legal challenges not faced by heterosexuals. This leads to difficulties they face to access HIV prevention services such as VCT and treatment. For this study, we sought to understand the sexual mixing of the sexual minorities and the general population by looking at the molecular relationships of HIV-1 isolates in the two populations.

**Objectives:** 1) To determine the HIV-1 circulating subtypes amongst sexual minorities in Zambia”; 2) To compare the similarities of HIV-1 isolates between sexual minorities and the general populations; 3) To describe the resistance mutations in HIV-1 isolates from in the HIV-1 pol region in isolates from sexual minorities.

**Methods:** DBS samples from LGBT and general populations were collected between September 2012 and January 2013. Nucleic acid extraction from the punched out DBS was performed using the NucliSENS EasyQ HIV-1 assay EasyQ HIV-1 analyzer platform. PCR was conducted using the primers published byYang et al (2010). One step RT-PCR was performed on the extracted viral RNA for the production of viral cDNA of the HIV-1 pol gene as described by Yang C, McNulty A et al (2010). PCR products for the sequencing reaction were purified using the QIAQUICK PCR purification kit. Generated sequences were edited and assembled using the Sequencer version 5 editing tool and analyzed using the Stanford HIVDB. Subtype identities were determined using the REGA subtyping tool.

**Results and Discussions:** Mean viral load was 1353copies/ml (400-16,666 copies/ml). 93 out 120(81LGBTs + 39 general population) had a viral load ≥ 400 copies/ml. 19 HIV-1 pol were successfully amplified (20.4 %). 17 HIV-1 pol successfully sequenced (6 general population & 11LGBTs). All study sequences (*pol* gene) clustered with HIV-1 subtype C. The study sequences did not clustered together but mixed with those of the general population.

No study sequence harbored high-level or intermediate resistance to NRTIs. One study sequence harbored resistance mutation (K103N) to EFV and NVP. Three from the General population harbored resistance to EFV and NVP, while two harbored intermediate resistance to RPV and ETR. Sexual minorities don’t live in isolation from the general populations. It is now recognized that they can be a serious reservoir of the HIV that could compromise HIV control programs.They are therefore an important target population for HIV prevention.

**Conclusions:** That the study sequences did not clustered own their own indicates high levels of bi-sexual relations among the so called sexual minorities who don’t access the provided for HIV service program for the general public for various reasons. Specific strategies should be developed for this population in order to curb the HIV-1 reservoir problem.

### Theme 3: Capacity Building for HIV Prevention and Vaccine Research – CapB

#### P9 The Development of HIV Bio-bank resource management to support clinical trial and Intervention research: WAPHIR experience

##### Moussa Thiam^1^, Diabou Diagne-Gueye^1^, Mame K. Ndiaye^1^, Moustapha Mbow^1^, Birahim P. Ndiaye^1^, Ibrahima Traore^2^, Mamadou C. Dia^2^, Gilleh Thomas^3^, Coumba Tour-Kane^1^, Souleymane Mboup^1^, Assan Jaye^3^

###### ^1^Laboratory of Bacteriology-Virology, Le Dantec Hospital of Dakar, Senegal and Cheikh Anta DIOP University of Dakar, Dakar, Senegal, ^2^Social Hygiene Institute of Dakar, Dakar, Senegal, ^3^Medical Research Council, Fajara Unit, Banjul, The Gambia

####### **Correspondence:** Moussa Thiam (Thiammoussa81@gmail.com) – Laboratory of Bacteriology-Virology, Le Dantec Hospital of Dakar, Senegal and Cheikh Anta DIOP University of Dakar, Senegal

**Background:** The establishment of the West African Platform for HIV Intervention Research (WAPHIR) in 2010 required the unification and management of HIV cohort resources in Senegal and from our partner institutions in the Gambia and Guinea Bissau. The setup of the Senegal WAPHIR Bio-bank supported by a unified database system is important for supporting planned collaborative intervention research and HIV clinical trial.

**Methods:** A quality management system (QMS) following the NF S 96-900 and ISO 15189 standards was established to define the Biobank policy. The Biobank processes were identified using a process approach according to the ISO 9001 v.2008. The linkage between those processes and their feasibility were investigated by performing a Biobank pilot study. Processed samples were logged, stored then, if needed, disseminated to users by a dedicated software (Item Tracker) and the generated database was conditionally shared using a Structured Query Language server.

**Results and Discussions:** The implanted QMS facilitated the Biobank activities by written forms (n = 12) and standard operational procedures (n = 9). The identified processes (n = 10) were linked through a process mapping with a documentation and communication system. The Biobank approved protocol was conducted using appropriate facilities. Biobank resources were collected in two cohorts and the generated database contains information from HIV-1 and HIV-2 infected patients aged ≥ 18 years. For the female sex workers cohort from the Social Hygiene Institute, we enrolled 817 patients (754 seronegative and 63 seropositive). A number of 1399 samples (plasma, Buffy coats and paxgen) and 297 PBMC samples were logged and stored respectively in -80 °C freezer and in liquid nitrogen tank. For the serodiscordant cohort at Fann hospital, fifty five serodiscordant couples were recruited. Samples were logged stored in -80 °C freezer and in liquid nitrogen tank respectively for 882 samples (plasma and Buffy coat and 147 paxgen) and for 729 PBMC.

**Conclusions:** This organized repository with a secured database is an institutional resource that could serve as a key for good quality research and to support clinical trials. However, the Biobank activities should be supported by new HIV projects that will strengthen the HIV platform and attract international collaboration.

#### P10 Capacity building for clinical trials as a novel approach for scaling up HIV prevention research initiatives in East Africa: achievements and challenges

##### Emily Nyanzi^1^, Edward Katongole Mbidde^1^, Pontiano Kaleebu^1,2^, Juliet Mpendo^3^, Joshua Kimani^4^ Josephine Birungi^5^, Winnie Muyindike^6^, Andrew Kambugu^7^

###### ^1^Uganda Virus Research Institute, Entebbe, Uganda; ^2^MRC/UVRI Uganda Research Unit on AIDS, Entebbe, Uganda; ^3^UVRI-International Aids Vaccine Initiative, Entebbe, Uganda; ^4^Kenya AIDS Control Project, University of Nairobi, Nairobi, Kenya; ^5^The Aids Support Organization (TASO), Kampala, Uganda; ^6^Mbarara University of Science and Technology, Mbarara, Uganda; ^7^Infectious Disease Institute, Mulago, Kampala Uganda

####### **Correspondence:** Emily Nyanzi (enyanzi@uvri.go.ug) – Uganda Virus Research Institute, Entebbe, Uganda

**Background:** Canada Africa Prevention Trials Network (CAPT N) initiated a program in 2010 to create and strengthen sustainable leadership capacity for African sites to conduct HIV Prevention Trials, promote collaborative South to South and North South Multidisciplinary HIV Research initiatives. The Network has 8 sites in Africa and 7 collaborating institutions in Canada.

**Methods:** The Network’s steering committee gives policy and advisory services on research and mentorship through regular teleconferences. Scientists in Africa and Canada have worked together on research projects as supervisors and mentors. CAPTN provides appropriate mentorship to graduates of Masters/ PhD and health practitioners with hands on learning experience in HIV research. The mentor must provide formal experiential learning environment including the development and management of at least one HIV research project for the mentee.

**Results and Discussions:** The Network funded circumcision and high incidence studies on HIV Infection in fishing communities around Lake Victoria, Uganda. The studies contributed evidence to policy to the regional ministries of health on treatment as prevention and ABCC strategy for HIV prevention. Capacity for seven MSc (2011), one Ph.D. (2012) and seven mentorships (2014) was built. In 2011 a six teams Grant on Violence, Gender and Health, worth $8.5 M for researchers and knowledge users at all sites was supported. In March 2012, CAPT-N hosted an Afri-Can forum 1.0 for over 300 HIV researchers from Africa and Canada to re-think strategies in HIV clinical and vaccine research. Three young scientists were awarded for their impressive carrier growth, with two of them coming from East Africa. Research intervention studies have created awareness resulting in increased number of people on ART and reduced HIV related deaths. Salary support provided to institutions facilitated the institutionalization of research in organizations that were initially service delivery orientated. Challenges include rampant concurrent sexual relationships leading to new infections. Also, turnover of scientists to NGOs pose a challenge to timely implementation of project activities.

**Conclusions:** There is need to continue complementing, promoting, strengthening and supporting CAPTN sites in building capacity for the existing experience and expertise in research between Africa and Canada through trainings and coordination of research activities.

#### P11 Community and media perspective of research; an advocacy workshop on HIV prevention research

##### Hachizovu Sebastian, Handema Ray, Chaponda Mike, Kabuya Jean Bertin, Mulenga Modest

###### Tropical Disease Research Centre, Ndola, Zambia

####### **Correspondence:** Hachizovu Sebastian (Sebastianhachizovu@yahoo.com) – Tropical Disease Research Centre, Ndola, Zambia

**Background:** HIV is a major health problem globally though Sub Saharan Africa is the worst hit. In 2012, there were 35.3 million people infected with the virus with more than 75 % being in Sub Saharan Africa. Additionally, 2.3 million new infections occurred in the same year. As a strategy to address the problem of HIV, a lot of countries are focusing their efforts on prevention. This saw 26 countries recording 50 % reduction in new infections between 2001 and 2012, while others lagged behind on this score. In order to achieve the desired results on the prevention front, all stakeholders need to be fully involved and to double their efforts. As an initiative to build Capacity for HIV Prevention Research, the TanZamBo organized a two days workshop in Zambia as part of the advocacy of the TDRC on HIV Prevention Research.

**Methods:** A workshop was organized to discuss issues related to HIV prevention research and the role of the community. Participants included; community leaders, ordinary community members, community health workers, community lay Counselors, staff from the print and electronic media and sexual minorities. Participants were taken through modules, given exercises, group discussions on specific topics and presentations in plenary.

**Results and Discussions:** Communities perceive research to be a money spinner, associated with Witchcraft and Satanism. It is a waste of time as there is no feedback given to the communities. Communities are reluctant to participate in research due to ignorance, no incentives, monotonous questions and lack of community involvement at inception. These challenges can be addressed by; setting Community Advisory Committees, proper community engagement and researching on community generated problems. Solutions lay in engaging community leaders early, knowing the community contexts and giving feedback on results. Additionally, the media should do community sensitization before, during and after the research and also results dissemination.

**Conclusions:** Research is a very important tool in HIV prevention. However, the community should be involved from the start for them to have ownership of the research. The media can play a very important role in sensitizing people on research and dissemination of results.

### Theme 4: HIV Treatment and Clinical Intervention – TxCln

#### P12 Development of a quantitative HIV-1 and HIV-2 real time PCR (qRT-PCR) viral load assay

##### Moussa Thiam^1^, Omar Janha^2^, Alberta Davis^2^, Alfred Amambua-Ngwa^2^, Davis C. Nwakanma^2^, Souleymane Mboup^1^, Assan Jaye^1,2^

###### ^1^Bacteriology and Virology Laboratory, Le Dantec Hospital of Dakar, Senegal and Cheikh Anta DIOP University of Dakar, Dakar, Senegal; ^2^Medical Research Council, Fajara Unit, Serrekunda, The Gambia

####### **Correspondence:** Moussa Thiam (Thiammoussa81@gmail.com) – Bacteriology and Virology Laboratory, Le Dantec Hospital of Dakar, Senegal and Cheikh Anta DIOP University of Dakar, Senegal

**Background:** The measurement of HIV viral load (VL) in a feasible and cheap format is important for efficient monitoring of antiretroviral therapy (ART) and for understanding HIV pathogenesis before and after treatment. HIV VL measurement continues to pose a challenge because available HIV-1 VL assays are expensive to use in resource-limited settings and there is no commercially validated HIV-2 viral load (VL) assay. The aim of this study is to develop an accessible HIV-1 and HIV-2 qRT-PCR assay for the virological monitoring of HIV infected patients in West Africa.

**Methods:** HIV-1 and HIV-2 primers and probes were designed targeting the *ltr* gene using NCBI blast and Primer3, a free online primer design tool. RNA extraction was done using the QIAamp Viral RNA Mini kit on 200 μl (HIV-1) and 300 μl (HIV-2) of plasma. The TaqMan Reverse Transcription and TaqMan Universal master Mix II with UNG were used respectively for the reverse transcription and the real time PCR with the ABI 7500 real time PCR machine.

**Results and Discussions:** The optimized primers and probe concentrations were respectively 0.3 and 0.5 μM (HIV-1) and 0.15 and 0.5 μM (HIV-2). The HIV-1 and HIV-2 qRT-PCR analytical specificities were determined by amplifying the expected products, which were 86 and 75 base pairs respectively for HIV-1 and HIV-2, without nonspecific targets. The developed qRT-PCR assay had a dynamic range of 10^6^ to 50 copies. The analytical sensitivity (low limit of detection) was estimated at 50 copies both for HIV-1 and HIV-2. Amplification efficiencies were obtained from the different standard curves and corresponding to PCR efficiencies at 90 % (HIV-1) and 99 % (HIV-2). Characteristics of the accepted standard curves for HIV-1 and HIV-2 qRT-PCR assays were: slope (-3.70 to -3.34.) and good linear correlation (R2 ≥ 0.992).

**Conclusions:** Optimisation of primer and probe concentrations along with amplication standisation have permitted ideal standard curves for copy numbers of HIV-1 and HIV-2 in a qRT-PCR assay. Other performance characteristics such as clinical sensitivity and specificity then reproducibility, will be determined for the assay validation.

#### P13 Differential effects of sex in a West African Cohort of HIV-1, HIV-2 and HIV-1/2 dual infected patients: Men are worse off

##### Sanne Jespersen^1,2^, Bo Langhoff Hønge^1,2,6^, Joakim Esbjörnsson^3,4^, Candida Medina^5^, David Da Silva TÉ^5^, Faustino Gomes Correira^5^, Alex Lund Laursen^2^, Lars Østergaard^2^, Andreas Andersen^1^, Peter Aaby^1,6^, Christian Erikstrup^7^, Christian Wejse^1,2,8^, for the Bissau HIV Cohort study group

###### ^1^Bandim Health Project, Indepth Network, Bissau, Guinea-Bissau; ^2^Department of Infectious Diseases, Aarhus University Hospital, Aarhus, Denmark; ^3^Department of Experimental Medical Science, Section of Molecular Virology, Lund University, Lund, Sweden; ^4^Weatherall Institute of Molecular Medicine, University of Oxford, Oxford, United Kingdom; ^5^National HIV Programme, Ministry of Health, Bissau, Guinea-Bissau; ^6^Research Center for Vitamins and Vaccines (CVIVA), Bandim Health Project, Statens Serum Institut, Copenhagen, Denmark; ^7^Department of Clinical Immunology, Aarhus University Hospital, Aarhus, Denmark; ^8^GloHAU, Center for Global Health, School of Public Health, Aarhus University, Aarhus, Denmark

####### **Correspondence:** Sanne Jespersen (Sanne.jespersen@clin.au.dk) – Bandim Health Project, Indepth Network, Bissau, Guinea-Bissau

**Background:** A disproportionate number of women in Sub-Saharan Africa are infected with HIV but the proportion of men enrolled in antiretroviral treatment programs is lower than the proportion of women. Several studies have reported conflicting effects of sex on HIV-1 infection. We describe differences in baseline characteristics and assess the impact of sex on HIV progression among patients at a clinic with many HIV-2 and dually infected patients.

**Methods:** The study utilized a retrospective cohort of treatment-naïve adults at the largest HIV clinic in Guinea-Bissau from June 6, 2005, to December 1, 2013. Baseline characteristics were assessed and the patients followed until death, transfer, loss to follow-up, or June 1, 2014. We estimated the time from the first clinic visit until initiation of ART, death, or loss to follow-up using Cox proportional hazard models.

**Results and Discussions:** A total of 5,694 patients were included in the study, 3,702 women (65 %) and 1,992 men (35 %). Women were more likely than men to be infected with HIV-2 (19 % vs. 15 %, p < 0.01) or dually infected with HIV-1/2 (11 % vs. 9 %, p = 0.02). For all HIV types, women were younger (median 35 vs. 40 years), less likely to have schooling (55 % vs. 77 %) or to be married (46 % vs. 67 %), and had higher baseline CD4 cell counts (median 214 vs. 178 cells/μl). Men had a higher age-adjusted mortality rate (hazard rate ratio (HRR) 1.29, 95 % confidence interval (CI) 1.09-1.52) and were more often lost to follow-up (HRR 1.27, 95 % CI 1.17-1.39).

**Conclusions:** Significant differences exist between HIV-infected men and women regardless of HIV type. Men seek treatment at a later stage and, despite better socioeconomic status, have higher mortality and loss to follow-up than women. This study demonstrates the clear need for increased attention to undiagnosed male HIV patients in Africa, as they are less likely to seek treatment, putting both their own lives and others’ at risk. Promoting earlier initiation of HIV treatment among men may improve their outcomes.

#### P14 HIV-infected adolescents in transition from pediatric to adult HIV care in Dakar, Senegal: sample characteristics and immunological and virological profiles

##### Siry Dieye^1,2^, Moussa Sarr^2, 3^, Haby Sy^4^, Helene D Mbodj^4^, Marianne Ndiaye^1^, Amy Ndiaye^1^, Seydi Moussa^1^, Assan Jaye^2, 5^, Souleymane Mboup^2^

###### ^1^Centre de Recherche Clinique et de Formation (CRCF) Infectious Diseases Dept, Fann Hospital, Dakar, Sénégal; ^2^Laboratory of Bacteriology and Virology, Le Dantec Hospital, Dakar, Senegal; ^3^Westat, Rockville, MD, USA; ^4^Albert Royer Children’s Hospital, Fann Hospital, Dakar, Senegal; ^5^Medical Research Council (MRC), Serrekunda, The Gambia

####### **Correspondence:** Siry Dieye (sirybaba@yahoo.fr) – Centre de Recherche Clinique et de Formation (CRCF) Infectious Diseases Dept, Fann Hospital, Dakar, Sénégal

**Background:** An increasing number of HIV infected adolescents are being transferred from pediatric centers to adult HIV-care. We currently do not have much information about this population in low prevalence countries in Africa, such as in Senegal. Our objective was to describe the demographical, clinical, immunological and virological status of infected adolescents in transition to adult HIV care in Dakar.

**Methods:** We used for these analyses follow-up data of HIV-infected adolescents in transition to adult HIV-care at the Fann Hospital’s “Center of Clinical Research and Training” in Dakar, Senegal. Descriptive analysis, generalized estimating equation (GEE) and mixed models were used to assess immunological and virological changes from baseline to month 24.

**Results and Discussions:** Twenty-nine infected adolescents were transitioned since 2011. The median (IQR) of age was 18 years (IQR = 2; min = 15- max =22), and 69 % were male. At baseline or entry, the median CD4 T-cell count was 478/μL (IQR = 296) and 28 % had prior CDC category C events. The median Log10 viral load was 3.72 (IQR = 1.22)) and 34 % had undetectable viremia at admission. The median time of exposure to ART was 6 years (range: 0 – 10 years). Forty one percent had a history of virologic failure and 55 % were on second line antiretroviral therapy.

During follow-up the median CD4 count went from 478 (IQR = 296) at entry to 490 (IQR = 274) at month 6, 532 (IQR = 552) at month12, 618 (IQR = 545) at month 18 and 721 (IQR = 550) at month 24, with a non-significant change the same period. Similarly, the viral load measures did not worsen during the transition period, with a mean Log10 viral load measure of 3.07 (STD = 1.41) at entry, 2.51 (STD = 1.11) at month 6, 2.51 (STD = 1.36) at month 12, 3.31 (STD = 1.45) at month 18, and 2.55 (STD = 1.47) at month 24.

**Conclusions:** Usually, it is expected that the quality of care will drop and be disturbed during the transition period of youth from pediatric to adult care. However, in our clinics at the Fann hospital in Senegal, the transition and follow-up process can be seen as successful, with the immunological and virological profiles of adolescents remaining the same during this sensitive period.

#### P15 Molecular characterization of vertically transmitted HIV-1 among children born to HIV-1 seropositive mothers in Northern Tanzania

##### Balthazar M. Nyombi, Elichilia R. Shao, Innocent B. Chilumba, Sikhulile Moyo, Simani Gaseitsiwe, Rosemary Musonda

###### Kilimanjaro Clinical Research Institute, Moshi, Tanzania

####### **Correspondence:** Balthazar M. Nyombi (b.nyombi@kcri.ac.tz) – Kilimanjaro Clinical Research Institute, Moshi, Tanzania

**Background:** HIV-1 is characterized by great genetic diversity due to mutations that occur during viral replication, drug and immunological pressures. The HIV-1 genetic diversity poses challenge and has implications on the pathogenesis, transmission, diagnosis, effective vaccine development and antiretroviral drug resistance development. The aims of this study were to characterize and determine mutations associated with antiretroviral drug resistance among HIV-1 infected children aged less than 18 months born to HIV-1 infected mothers enrolled in prevention of mother-to-child transmission services in Northern Tanzania.

**Methods:** Dried blood spots (DBS) were collected from children born to HIV-1 infected mothers for HIV-1 early infant diagnosis from January 2011 to December 2012. DBS were tested by PCR at Kilimanjaro Christian Medical Center (KCMC) Clinical Laboratory. Viral load was estimated and genotypic resistance testing was determined on samples with a viral load greater than 400 copies/mL.

**Results and Discussions:** A total of 640 DBS were collected and among them 122 were HIV-1 positive by PCR with the mean viral load of 387 copies/mL. The median age was 12 weeks (IQR 6-28). Among the positive samples that were eligible for genotyping, sequences were successfully generated from 46 samples. HIV-1 subtype A was predominant (27/46) followed by subtype C (10/46), subtype D (3/46), CRF10_CD (2/46) and unique recombinant forms (4/46). Genotypic resistance mutations were detected in 13 of 46 children (28 %). The prevalent mutations were Y181C ( 8/13) and K103N (4/13) associated with non-nucleoside reverse transcriptase inhibitors (NNRTI) because of their extensive use. Protease inhibitors are not extensively used and none associated mutation was detected in the protease region.

**Conclusions:** HIV-1 subtype A was more prevalent among vertically transmitted HIV-1 strains from mother to child as compared to other co-circulating HIV-1 subtypes in northern Tanzania. Mutations associated with resistance to non-nucleoside reverse transcriptase were more predominant due to extensive use of the drug in the prevention of mother to child HIV-1 transmission. Resistance to antiretroviral drug resistance testing may be warranted among children with vertically transmitted HIV-1 infection before starting highly active antiretroviral treatment.

#### P16 Breast-fed HIV-1 exposed infants play catch up. A preliminary report

##### Pam Datong^1,2^, Bucky Inyang^1^, Sophia Osawe^2^, Abel Izang^1^, Chundung Cole^1^, Felicia Okolo^2^, Bill Cameron^3^, Kenneth Rosenthal^4^, Clive Gray^5^, Heather Jaspan^5^, Alash’le Abimiku^2,6^, the INFANT study team

###### ^1^Plateau State Specialist Hospital, Jos, Nigeria; ^2^Plateau State Human Virology Research Center, Institute of Human Virology, Jos, Nigeria; ^3^Divisions of Infectious Diseases and Respirology, University of Ottawa at The Ottawa Hospital, Ottawa, Canada; ^4^Department of Pathology & Molecular Medicine, McMaster University, Hamilton, Ontario, Canada; ^5^Institute of Infectious Diseases and Molecular Medicine, Faculty of Health Sciences, University of Cape Town, Cape Town, South Africa; ^6^Institute of Human Virology, University of Maryland School of Medicine, Baltimore, Maryland, USA

####### **Correspondence:** Pam Datong (pdatong@yahoo.com) – Plateau State Specialist Hospital, Nigeria

**Background:** Growth is a good indicator of the overall well being of a child. Growth failure or deceleration has been closely associated with increased health problems and sometimes death in infants. We reported earlier that infants born to HIV infected mothers are twice at risk of death mainly due to infectious morbidities. In this respect, growth monitoring particularly in HIV exposed children is an important tool to ensure timely and appropriate care for these children.

**Methods:** A cohort of HIV exposed (HIV-EU) marched with HIV unexposed (HIV-N) infants were enrolled at birth and followed up during 8 study visits in 52 weeks in Jos Nigeria. Baseline assessment consisted of gestational age, physical examination, anthropometrics, noting of gross congenital anomalies and birth complications. Newborns with birth complications were excluded. Anthropometrics, co-morbidities and feeding practices were evaluated at each study visit.

**Results and Discussions:** Two hundred and thirty six HIV-EU and 76 HIV-N infants were enrolled. At birth, HIV-EU infants were smaller in weight, length and head circumference than HIV-N infants (mean weight 2959 g, SD 398 vs. 3189 g, SD 529, p value 0.0001; mean length 47.1 cm, SD 3.13 vs. 48.6 cm, SD 3.69, p value 0.0005; and mean head circumference 34.3 cm, SD 1.59 vs. 35.2 cm, SD 1.72, p value 0.0001). Although, there was impaired weight gain and head circumference increment in HIV-EU compared with HIV-N infants, the length of HIV-EU caught up with that of HIV-N infants by 15 weeks. The proportion of HIV-EU that were exclusively breast-fed for the first 6 months was comparable to HIV-N infants (209/236, 88.56 % vs. 68/76, 89.66 % p value 0.826.

**Conclusions:** Our study seem to indicate that breastfeeding which is the usual practice in Nigeria could improve nutrition and correct linear growth faltering in infants. We plan to continue to assess developmental milestones to determine the consequences of early-impaired head circumference increment, weight and length observed in HIV-EU infants since growth faltering in early childhood has been associated with increased risks of glucose intolerance and cardiovascular diseases in adulthood.

#### P17 The frequency of N348I mutation in patient failing combination antiretroviral treatment In Botswana

##### Boitumelo Seraise^1,2^, Kerstin Andrea-Marobela^1^, Sikhulile Moyo^2^, Rosemary Musonda^2^, Joseph Makhema^2^, Max Essex^2, 3^, Simani Gaseitsiwe^2^

###### ^1^University of Botswana, Department of Biological Sciences, Gaborone, Botswana; ^2^Botswana Harvard AIDS Institute Partnership, Gaborone, Botswana; ^3^Harvard School of Public Health, Department of Immunology and Infectious Diseases, Boston, USA

####### **Correspondence:** Boitumelo Seraise (bseraise@bhp.org.bw) – University of Botswana, Department of Biological Sciences, Gaborone, Botswana

**Background:** The N348I mutation in the connection subdomain of HIV-1 Reverse Transcriptase (RT) has been reported to reduce susceptibility to Nevirapine (NVP), Efavirenz (EFV) and Zidovudine (AZT), and is frequently selected in HIV-1 patients experiencing virologic failure. The frequency of this mutation is under reported as most of the studies only report RT mutations up to position 335. We here sort to investigate the frequency of the N348I mutation in HIV-1 subtype C infected patients in Botswana failing different antiretroviral therapy regimens as there is no data on the prevalence of this mutation in patients failing cART in Botswana.

**Methods:** HIV-1 drug resistance genotyping was done on samples from two previous clinical trials conducted at Botswana Harvard Partnership investigating the efficacy of different ART regimens. Plasma samples from 43 patients who experienced virologic failure from the two studies were available for genotyping. Virologic failure was defined as confirmed plasma HIV-1 RNA greater than 400 copies/mL at 6 months post HAART initiation or any time after initial virologic suppression. A PCR assay covering position 348 of HIV-1 RT connection subdomain was optimized and used to amplify the region followed by big dye sequencing of the PCR product. The RT sequences were analyzed for the N348I resistance mutation using the Stanford HIV drug resistance database.

**Results and Discussions:** Thirty-four of the 43 (79.1 %) virologic failure samples were successfully genotyped. Amongst the 34, 9(26.5 %) were found to harbor the N348I mutation. The N348I mutation emerged in 10 % of patients failing Zidovudine (AZT) containing cART and 33.3 % of participants failing regimens containing Nevirapine (NVP) or Efavirenz (EFV).

**Conclusions:** We found a frequency of 26.5 % for N348I mutation among HIV-1C patients experiencing virologic failure in Botswana. The association of the N348I mutation with virologic failure in this population warrants further investigation but in this cohort it seemed to be more closely linked with NNRTI failure than with AZT failure. The kinetics of the mutation in relation to other RT mutations also needs to be investigated to better understand its impact.

